# A New Phylogenomic Approach For Quantifying Horizontal Gene Transfer Trends in Prokaryotes

**DOI:** 10.1038/s41598-020-62446-5

**Published:** 2020-07-24

**Authors:** Eliran Avni, Sagi Snir

**Affiliations:** Department of Evolutionary Biology, University of Haifa, Haifa, 31905 Israel

**Keywords:** Computational biology and bioinformatics, Phylogenetics

## Abstract

It is well established nowadays that among prokaryotes, various families of orthologous genes exhibit conflicting evolutionary history. A prime factor for this conflict is horizontal gene transfer (HGT) - the transfer of genetic material not via vertical descent. Thus, the prevalence of HGT is challenging the meaningfulness of the classical Tree of Life concept. Here we present a comprehensive study of HGT representing the entire prokaryotic world. We mainly rely on a novel analytic approach for analyzing an aggregate of gene histories, by means of the quartet plurality distribution (QPD) that we develop. Through the analysis of real and simulated data, QPD is used to reveal evidence of a barrier against HGT, separating the archaea from the bacteria and making HGT between the two domains, in general, quite rare. In contrast, bacteria’s confined HGT is substantially more frequent than archaea’s. Our approach also reveals that despite intensive HGT, a strong tree-like signal can be extracted, corroborating several previous works. Thus, QPD, which enables one to analytically combine information from an aggregate of gene trees, can be used for understanding patterns and rates of HGT in prokaryotes, as well as for validating or refuting models of horizontal genetic transfers and evolution in general.

## Introduction

Deciphering the history of life on Earth is among the most fundamental and ancient task in biology. With the advent of high throughput sequencing technology, the belief of advancing in this seminal challenge was apparent and imperative. A new area called phylogenomics^1,2^ which integrates between genome analysis and systematic studies, was opened. A basic and ubiquitous step in phylogenomic studies is the inference of an ancestor-descendant relationship in the form a tree, dubbed as a *gene tree*, for every family of genes in a dataset. These studies have found widespread disagreements between these gene trees^3^, leading some to doubt the relevance of the Tree of Life concept^4–9^. These are not merely statistical erros. Rather, events such as duplications and losses in gene families, incomplete lineage sorting, and horizontal genetic transfers lead to conflicting gene histories^10–12^. It therefore seems that the abundance of data only makes deciphering the history of life on Earth a harder task than initially thought.

Here we study specifically horizontal gene transfer (HGT), that is, the transfer of genes between contemporaneous organisms not via vertical parent-offspring descent. Primarily, HGT is mediated by plasmids, transposons and other mobile elements, and viruses (bacteriophages). HGT tangles the conventional universal Tree of Life, turning it into a network of Evolution^4,6,7,9^. HGT is pervasive and some estimates of the genes undergone HGT amount to 99%, see e.g.^3,13^. In particular, HGT is common in prokaryotes and plays an important role in adaptation to new niches^14^.

Nevertheless, the belief in a species tree, underlying the major trend of organismal evolution still attracts researchers to resolve these entangled histories^15–17^. In particular, there is ample evidence that a strong tree-like signal can be extracted, even in the presence of extensive HGT. These results often rely on a blend of phylogenetic signals, including pairwise gene distances, a comparison to a reference species tree, theoretical studies, and the analysis of simulative and empirical data^3,18–23^.

Normally, prokaryotic history is constructed through inferring histories for ubiquitous genes that are believed to be immune to HGT, such as ribosomal RNA genes. However, such genes are also liable to HGT, hiding this vertical signal^24–27^. Moreover, for their housekeeping role these genes are under high conservation, and do not provide a rich enough evolutionary signal that is mandatory when classifying the deep branches of the tree of life. Therefore, it was proposed to consider a multitude of gene trees and subsequently amalgamate them into a unified tree encompassing all taxa under study, an approach denoted as the *supertree* operation^28,29^.

In this paper we follow up on previous works from E. Koonin’s group^18–20,30,31^. Consequently, some questions studied in these works are revisited, and some conclusions are reaffirmed. In the first part of the work we tackle the subjects outlined above where our results are derived primarily from a novel concept, the *quartet plurality distribution* (QPD, defined below), that was first introduced in^32^ and studied further in^33–35^. allowing us to indirectly obtain two separate results in the realm of HGT research. A rigorous analysis of 6901 gene trees from a representative set of one hundred taxa including twenty two phyla and approximating the entire prokaryotic world spectrum, reveals a strong bias towards bacteria’s confined HGTs (i.e. involving two bacteria). HGT is substantially less frequent when confined to archaea, and relatively rare when it involves one bacterium and one archaeon. This implies the existence of a barrier between the archaea and the bacteria, hindering successful HGT events between the two domains. Statistical computations show that these differences in HGT frequency are highly significant.

Despite the prevalence of HGT events within the bacteria (and to a lesser extent, within the archaea), our calculations show that a strong tree signal of evolution does exist. Accordingly, in the second part of the paper we construct two phylogenies that suggest a better compliance with several evolutionary evidences and criteria, compared to other hypothesized trees constructed before.

## Results

### An inter-domain HGT barrier

This part of the work focuses on analyzing the plurality quartets (defined below) derived from a collection of 6901 prokaryotic genes based on 41 archaea and 59 bacteria *Quartet Plurality Distribution*. (QPD), a concept that was introduced in^32^ and is further developed here we introduce and develop, is used to show that in the collection of genes that we analyze, HGT events are divided into three major categories: (1) Bacteria’s confined HGTs (involving two bacteria), that are most common; (2) Archaea’s confined HGTs, that are moderately common; and (3) HGTs between archaea and bacteria (Inter-domain), that are least common. In addition, simulations of biased HGT, as well as an and an independent analysis of the real data gene trees, are used to strengthen these results. QPD is defined succinctly in the Methods section. Here, a more detailed exposition that also discusses the rationale for the study of the plurality quartets and the QPD is given. We remark that the notion of quartet plurality (and the related triplet plurality) were used in several studies in the past, either for phylogenetic reconstruction^36,37^, or for identifying non-tree-like evolution^38–40^.

As evolution is time driven, it is perceived as directed - from ancestral root to descendants. However, due to lack of time indication in molecular sequences, gene histories (trees) are normally inferred unrooted. In such trees, the *quartet*, an unrooted tree over four leaves (species) is the minimal tree that carries any phylogenetic information. It is easily seen (Section 5.1 below) that there are exactly three possible quartet topologies based on a given 4-taxon set {*a*, *b*, *c*, *d*}: *a*, *b*|*c*, *d*; *a*, *c*|*b*, *d*; *a*, *d*|*b*, *c*; and every tree induces exactly one of the three topologies (the case of a star topology of the quartet is ignored). When examining an array of gene trees and a given 4-taxon set, the number of gene trees satisfying each one of the three topologies is counted. The topology satisfied by the greatest number of gene trees is denoted the *plurality quartet* and intuitively reflects the strongest tree signal supported by those genes. This procedure is referred to as *the plurality inference rule*, and is used to infer the plurality quartets of the 4-taxon sets under examination. It is noteworthy that according to the results of  ^41^, the reliability of the plurality quartet topology increases with the number of genes taken under consideration. Hence, all gene trees were incorporated in the computation of the plurality quartets, including individual genes that weakly support the quartet topology of some of the 4-taxon sets.

We use the term *plurality score* to denote the percentage of votes a plurality quartet attains and we note that the “plurality votes” may very well differ between the quartets. For example, while the topology *q* = *a*, *b*|*c*, *d* may be satisfied by 60% of the gene trees, another $q{\prime} =a,b| c,e$ may be satisfied by 90% of the gene trees. In the example above, *q* = *a*, *b*|*c*, *d* and $q{\prime} =a,b| c,e$ have 60% and 90% plurality scores respectively. When the plurality score of a given quartet is calculated, only gene trees in which the quartet is resolved are taken under consideration, while trees where the quartet is absent or unresolved are ignored. We denote by the *quartet plurality distribution*, or QPD the distribution of the plurality vote over a large set of quartets, all inferred from the same gene-tree set. Evidently, a strong tree signal is resulted in a high plurality score. Hence, gene trees with wide overall agreement will induce many plurality quartets with high plurality scores. Thus, we can exploit the plurality quartets as building blocks of the entire species tree and their plurality score as a confidence indication to this quartet topology.

#### Initial analysis of the real data QPD

**Figure 1:**
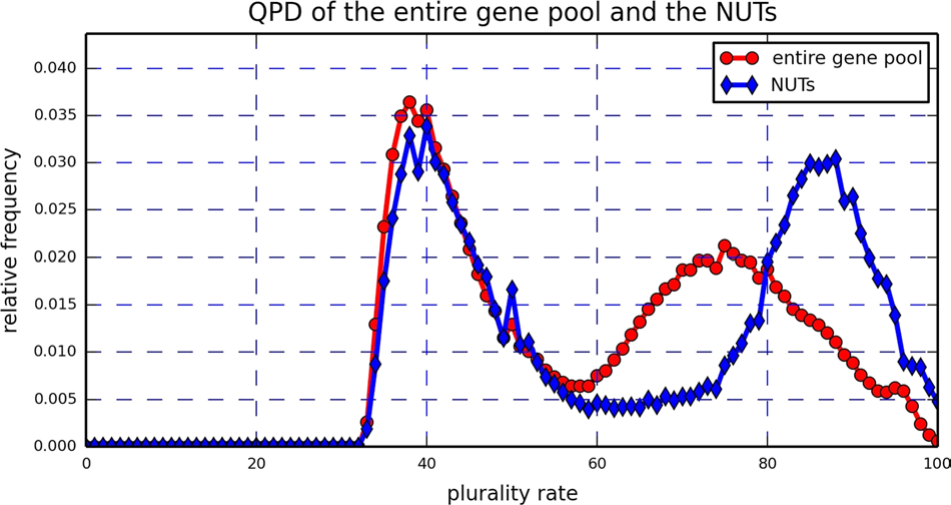
Real data QPDs, based on the entire gene pool (red circles) and on the NUTs (blue rhombuses). Two local maxima are clearly visible, a phenomenon which is absent from the QPDs pertaining to simulated data of uniform HGT. The higher values of the QPD based on the NUTs, compared to the QPD based on the entire gene pool, reinforce the claim that the NUTs are stabler than the genes in the entire gene pool.

**Figure 2:**
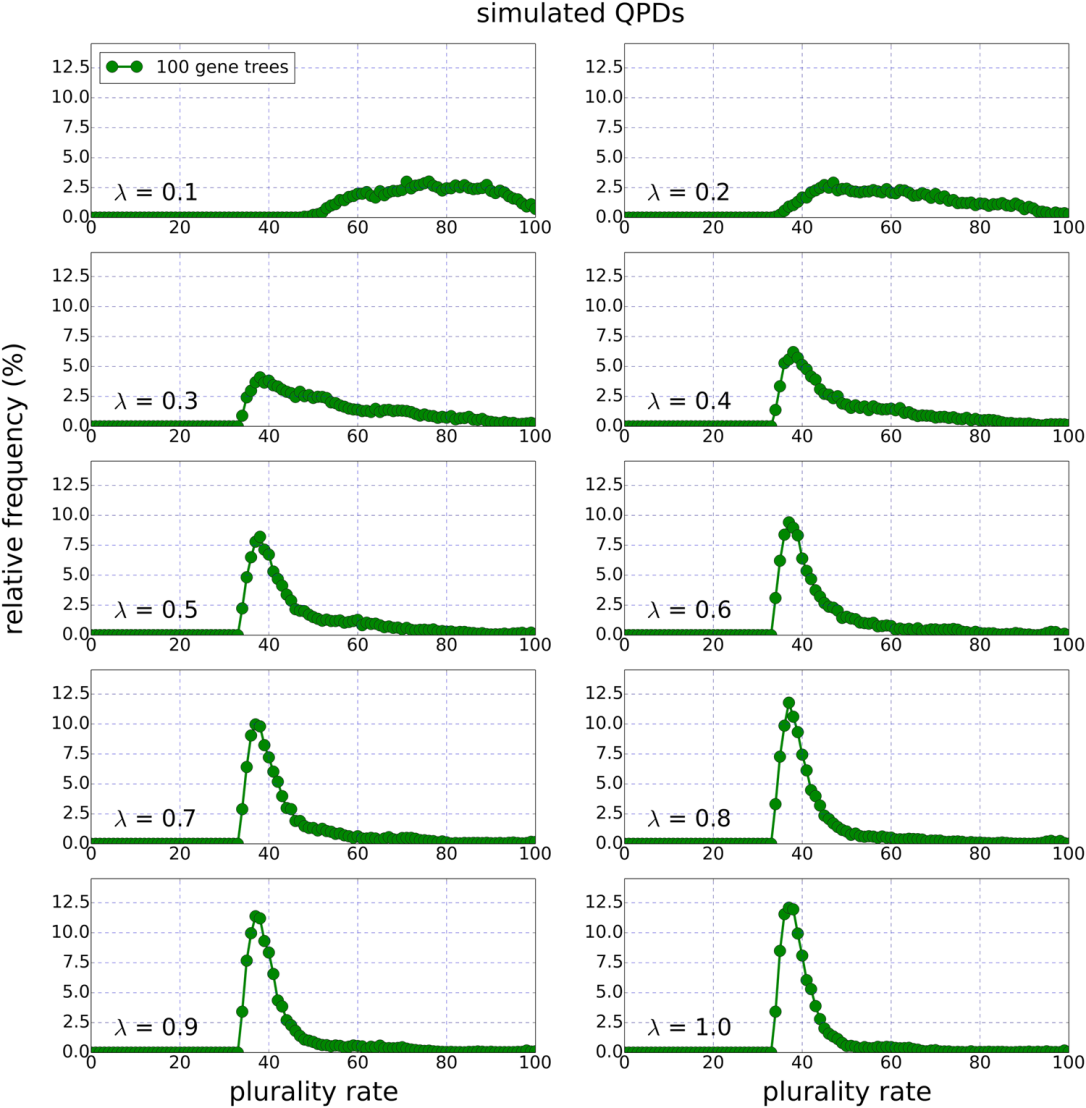
QPDs of the simulated data regarding a simulated species tree with *n* = 100 leaves and ten groups of simulated gene trees (where *λ* indicates the rate of HGT events in each group). Contrary to the real data QPDs, here only a single local maximum appears in each graph. The QPDs of this figure were computed based on collections of 10000 randomly sampled quartets. Similar graphs were produced for other simulated species trees and have shown a similar pattern (see S1 file, Appendix A).

The real data analysis includes a collection of 6901 gene trees of archaea and bacteria, with a total of 100 species (see Section 5.2 and the Table S1). Two collections of gene trees were examined, one is the entire gene trees pool, and the other is a set of 123 *nearly universal trees* (or NUTs), each consists of 90 taxa or more. We analyze the QPDs of the two collections of real data gene trees (Fig. 1) and compare it with the QPDs of ten groups of simulated gene trees composed of 100 taxa (Fig. 2). The ten groups of simulated genes trees, each composed of 100 trees and constructed according to a uniform HGT model (also employed in several other papers^16,22,23,42,43^), differ by the HGT “rates” that underlie their construction (*λ* = 0.1, 0.2, …, 1.0). See Section 5.3 for more details on the simulation process.

First, Fig. 1 reveals that the NUTs induce more quartets with high plurality scores compared to the general gene pool, which reinforces the claim that the NUTs are generally more conserved and immune to HGT than the average gene tree^18,19^. More importantly, a stark difference between the real data and the simulated data is that the two local maxima present in Fig. 1 are absent from all of the QPDs in Fig. 2. Furthermore, QPDs that were produced for simulated species trees with *n* = 10, 20, …, 90 taxa displayed similar features to the ones of Fig. 2 when the trees were large enough (30 taxa or more. See S1 file, Appendix A). Clearly, the pattern of real data QPD is inconsistent with the pattern emerging from the simulated data, based on the uniform HGT model.

#### Further study of the real data QPD

**Figure 3:**
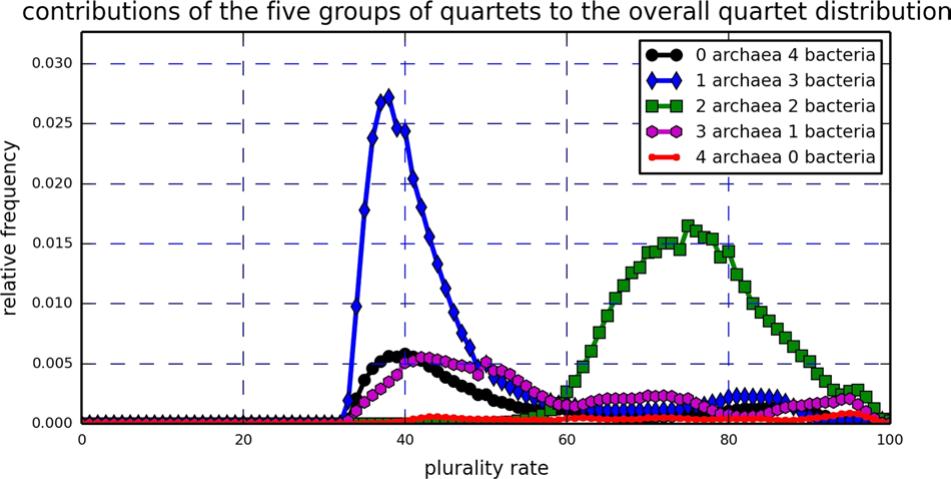
Real data: The contributions of five groups of quartets with 0,1,2,3 and 4 archaea (equivalently, 4,3,2,1 and 0 bacteria) to the real data QPD based on the entire gene pool. The contribution to the right peak of Fig. 1 comes primarily from quartets with 2 archaea and 2 bacteria, while the left peak of Fig. 1 comes from quartets with 3 bacteria or more. An equivalent calculation was carried out based on the NUTs and showed similar results. See S1 file, Appendix A.

In order to explain the bimodal structure of Fig. 1, the quartets were divided into five groups based on the number of archaea (equivalently, bacteria) they have: 0 archaea, 4 bacteria; 1 archaea, 3 bacteria; 2 archaea, 2 bacteria; 3 archaea, 1 bacteria; and 4 archaea, 0 bacteria. Then, the individual contributions of these groups to the overall QPD were plotted (Fig. 3). Evidently, the group comprised of quartets with 2 archaea and 2 bacteria is the main contributor to the right peak of the real data QPD, while the group comprised of quartets with 1 archaea and 3 bacteria creates the left peak. A direct examination of the quartets with 2 archaea and 2 bacteria showed that indeed an archaea-bacteria separation is induced by all 1,403,020 of them. Moreover, only 33 of these quartets, which is 8.41 × 10^−4^% of the entire set of induced quartets, had a plurality score of less than 50%. Contrary to that, when examining the remaining 2,518,205 quartets that are not comprised of 2 archaea and 2 bacteria, we saw that while roughly 1,580,000 of them, which is approximately 40.30% of the entire set of induced quartets, did not pass the 50% plurality score threshold. Comparing the quartets with 2 archaea and 2 bacteria to the remaining quartets using the Wilcoxon rank-sum test revealed that the difference between the two sets is extremely significant (Z-score greater than 1000). Thus, Fig. 3 suggests the existence of an archaea-bacteria barrier that results in a strong vertical tree signal separating the archaea from the bacteria and hinders successful HGT events between the two domains. However, the fact that most quartets with 2 archaea and 2 bacteria do not have a perfect (100%) plurality score suggests that such events do occur, as was previously reported^44,45^.

**Figure 4:**
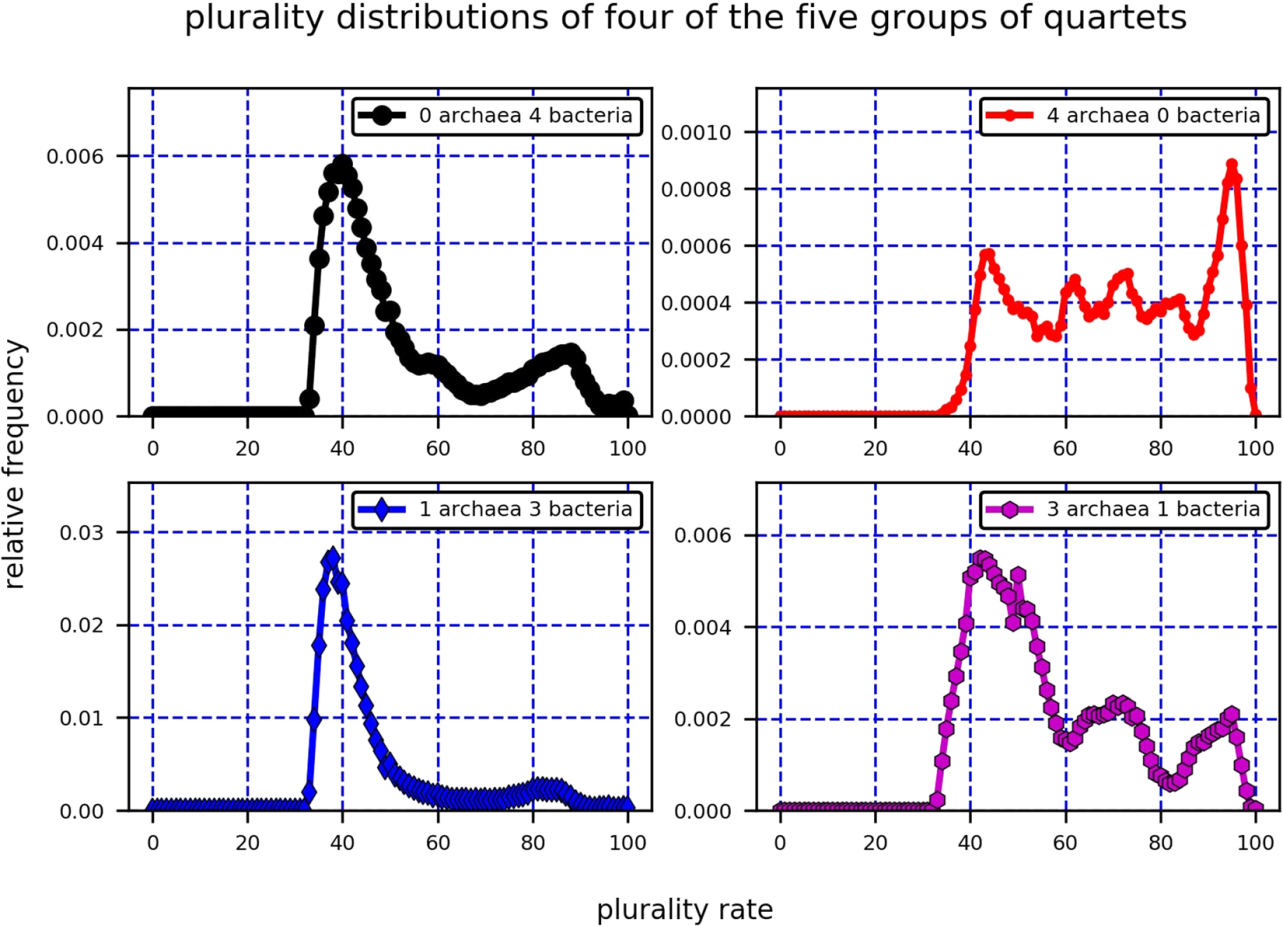
Real data: The QPDs of four of the five groups of quartets with 0, 1, 3, 4 archaea (equivalently, 4, 3, 1, 0 bacteria), when plotted independently, enable one to compare the probability of HGT events within the two domains. Low plurality scores of quartets with 3 or 4 bacteria compared to quartets with 3 or 4 archaea are suggestive that bacteria experience a relatively high number of HGT events among themselves.

In order to focus on the differences between the archaea and the bacteria, the contributions of four of the five groups to the overall QPD were plotted separately. Those four groups were: 0 archaea, 4 bacteria; 1 archaea, 3 bacteria; 3 archaea, 1 bacteria; and 4 archaea, 0 bacteria (Fig. 4). Indeed, this figure reveals that if a quartet has one or zero archaea (and three or four bacteria) then it is more likely to have a low plurality score, compared to a quartet with three or four archaea (and one or zero bacteria). Hence, Fig. 4 points to a difference between intra-bacteria HGTs and intra-archaea ones, the former being the more frequent of the two. Notice that despite the fact that intra-bacteria HGTs are more frequent than intra-archaea HGTs, quartets with 1 archaea and 3 bacteria, and not with 4 bacteria, form a majority among quartets with low plurality scores. This is a consequence of the number of archaea and bacteria involved in this study and the non-uniform HGT rates within and among the two domains, which is also demonstrated by the simulations of biased HGT conducted in this paper (Section 2.1.3). Interestingly, when comparing the proportion of quartets with 1 archaea and 3 bacteria that have low plurality rates to the proportion of quartets with 4 bacteria that have low plurality rates, no significant difference among the two sets is found (Fig. 4, left).

#### Corroboration via simulations of biased HGTs

**Figure 5:**
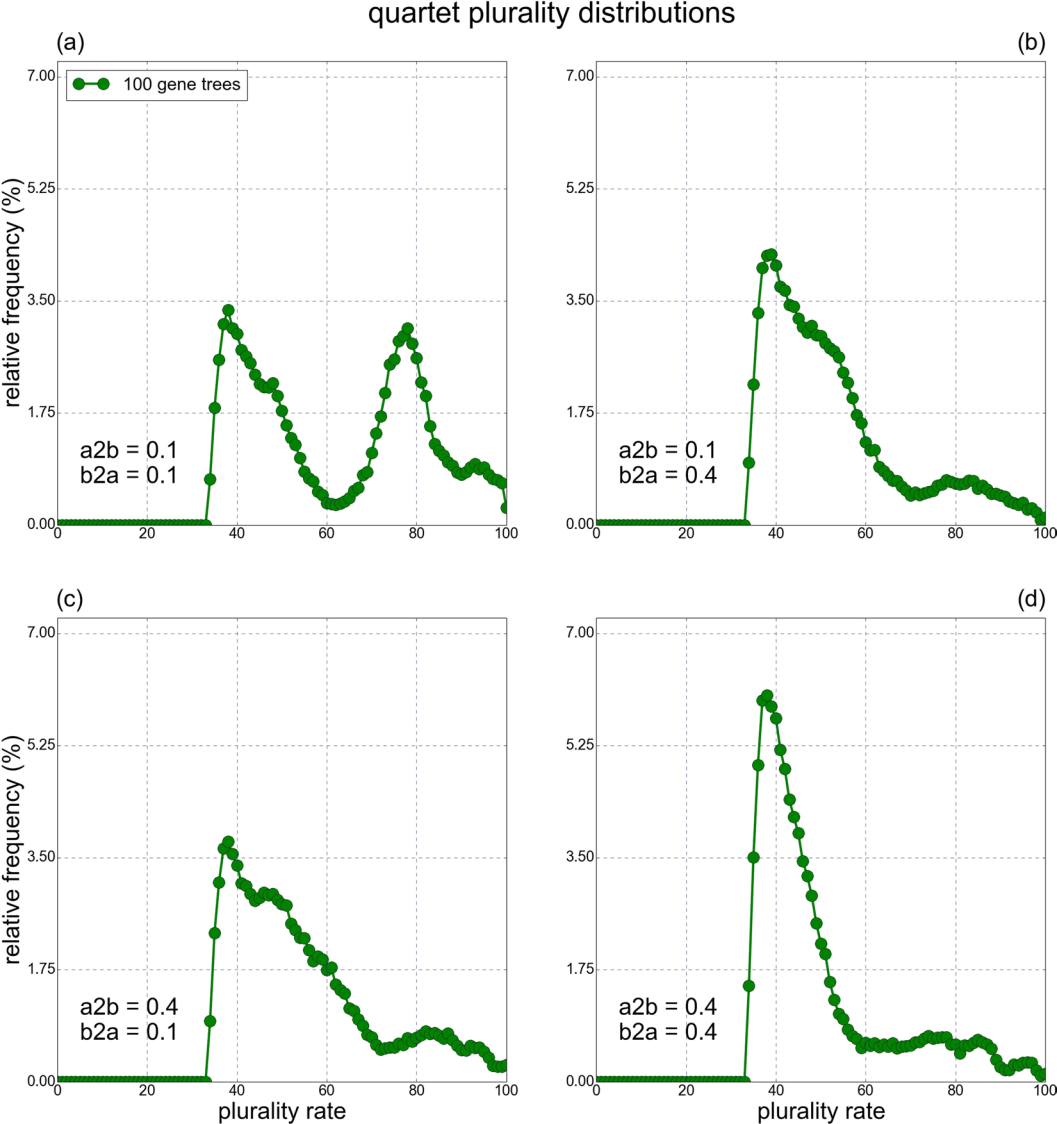
Examples of different simulated QPDs, generated using a biased HGT model, for an intra-archaea HGT rate of 0.6 and an intra-bacteria HGT rate of 0.8, with different inter-domain HGT rates. The HGT rates of archaea to bacteria (or bacteria to archaea) transfers are represented by *a*2*b* (or *b*2*a*). Only when *a*2*b* = 0.1 and *b*2*a* = 0.1 is a bimodal graph generated.

To corroborate the conclusions aforementioned, i.e. the existence of an HGT barrier (Fig. 3) and the greater prevalence of intra-bacteria HGTs compared to inter-archaea’s (Fig. 4), several scenarios of a simulation study based on a model of biased HGT events were tested in a simulation study: Five simulated species trees with 100 leaves were generated, and a node with 41 descendant leaves was identified. This node and all its descendants were defined as “archaea”. The remaining leaves and internal nodes were defined as “bacteria”. By varying intra-domain and inter-domain HGT rates we were able to simulate groups of gene trees with 100 trees in each group. Our results indicate that an inter-domain HGT rate which is at least twofold smaller than the intra-domain HGT rate is necessary for clear bimodal QPDs to be generated (for example, see Fig. 5). More details on the biased HGT model are found in Section 5.3.

**Figure 6:**
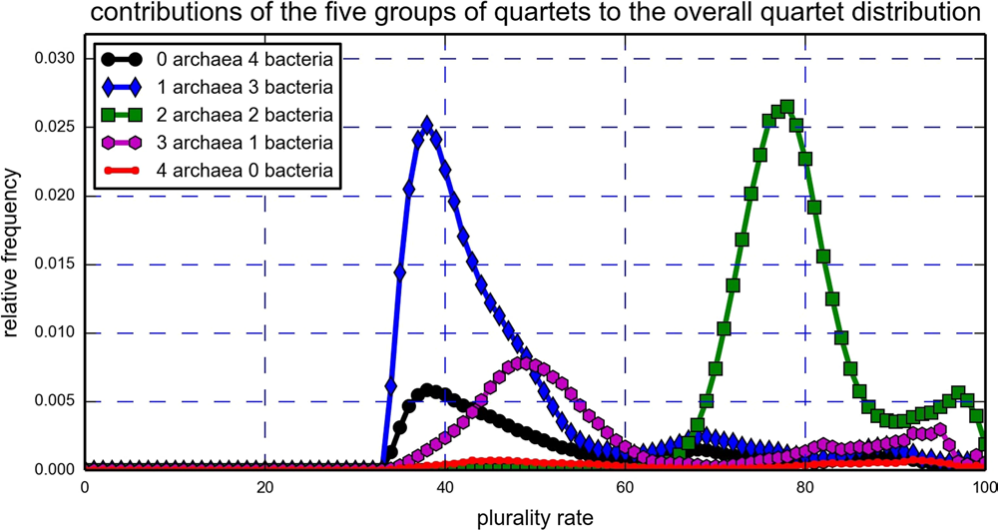
The contributions of the five groups of simulated plurality quartets (0 archaea 4 bacteria; 1 archaea 3 bacteria; 2 archaea 2 bacteria; 3 archaea 1 bacteria; 4 archaea 0 bacteria) to the simulated QPD of the entire quartets set reveal the source of the bimodal structure of the QPD. The data was generated using a biased HGT model, with an intra-archaea HGT rate of 0.6, intra-bacteria HGT rate of 0.8, and an inter-domain HGT rate of 0.1.

**Figure 7:**
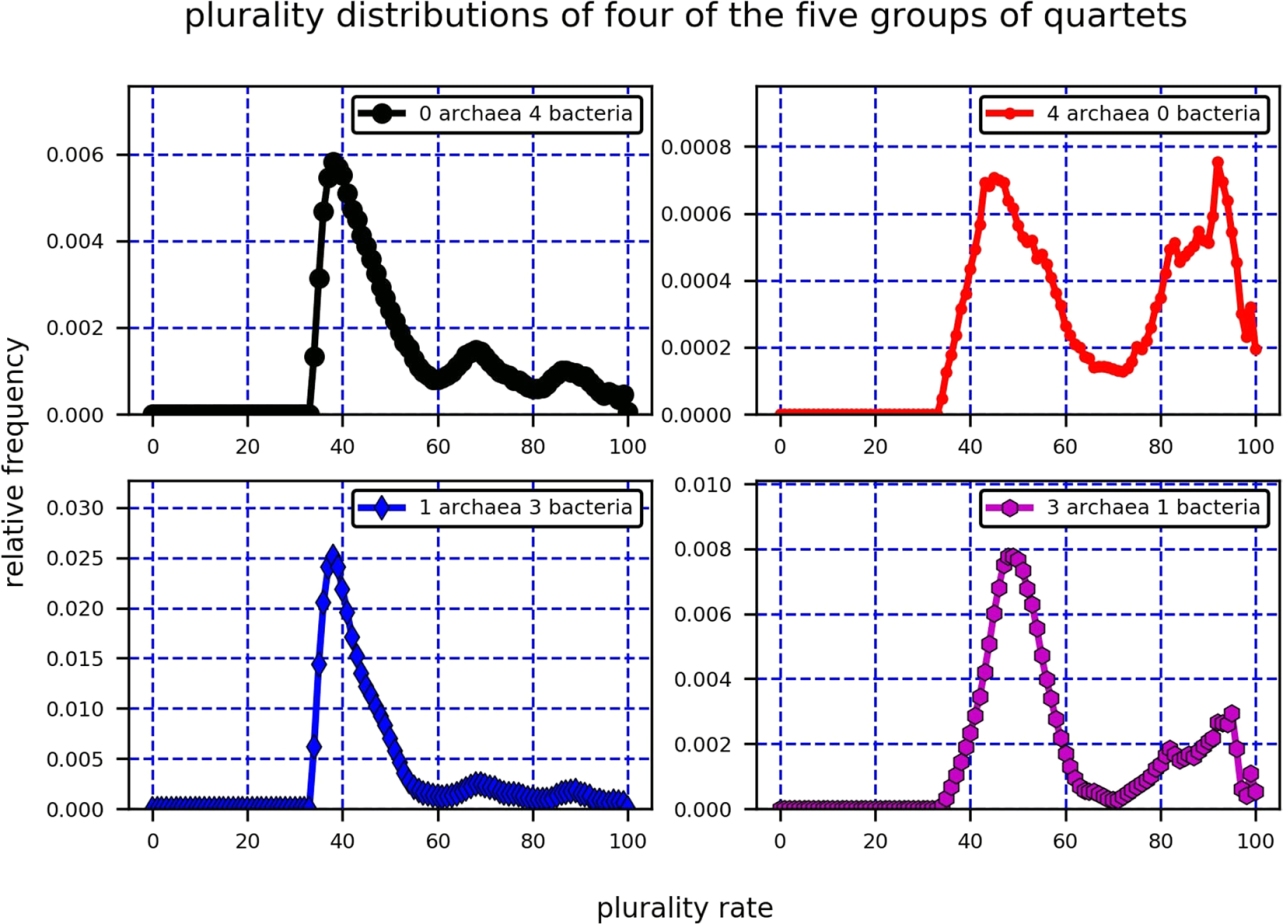
The QPDs of four of five groups of simulated plurality quartets (0 archaea 4 bacteria; 1 archaea 3 bacteria; 3 archaea 1 bacteria; 4 archaea 0 bacteria), plotted separately. Clear similarities exist between the simulated and the real data.

We further illustrate the similarity between the real data results and the biased HGT model. As in the real data analysis, the simulated plurality quartets pertaining to Fig. 5a were divided into five groups based on the number of archaea (or bacteria) each quartet has, and the contributions of each of the five groups to the QPD of the entire quartet set were plotted together (Fig. 6). In addition, the QPDs of four out of the five groups were plotted separately (all groups excluding quartets with 2 archaea and 2 bacteria, Fig. 7). Clearly, the simulated QPDs resemble the real data QPDs, as a bimodal structure is formed and quartets with 3 or 4 bacteria tend to have lower plurality scores compared to quartets with 3 or 4 archaea, according to the simulations and the real data. Interestingly, quartets with 2 archaea and 2 bacteria have a sharper peak in the simulated data than in the real data, suggesting that the HGT barrier between archaea and bacteria in nature is less restrictive than what the parameters pertaining to Figs. 6 and 7 dictate.

#### Independent study of the intra-domain HGTs

**Figure 8:**
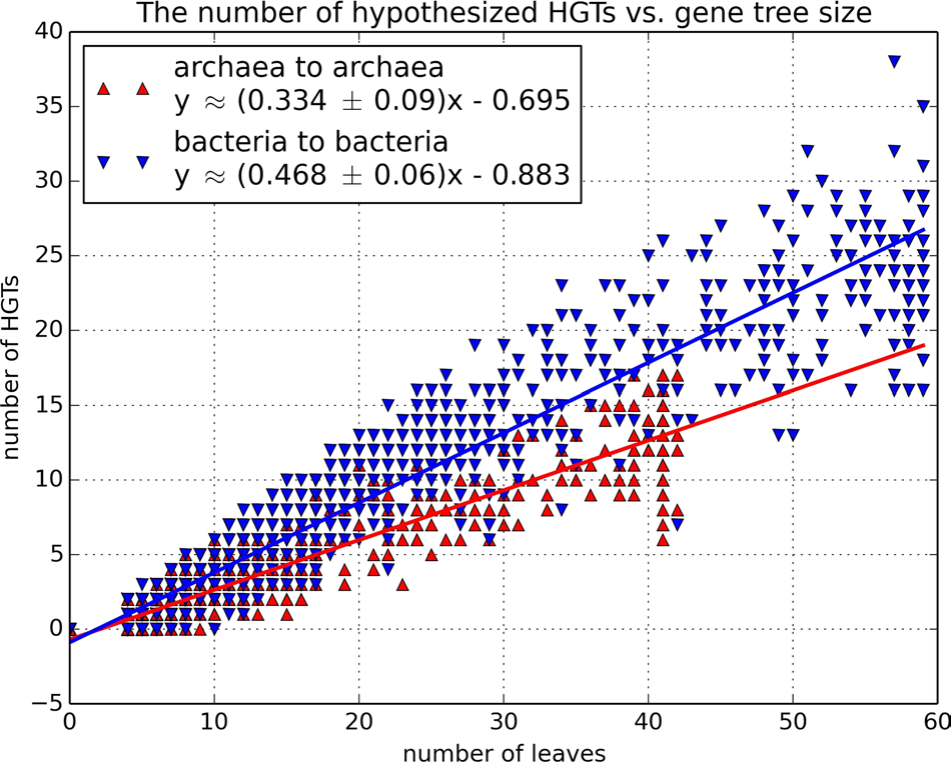
The number of HGTs computed by RIATA. The *y*-axis represents the SPR distance between the archaea-only gene trees (represented by red up-pointing triangles), or the bacteria-only gene trees (represented by blue down-pointing triangles) and the hypothesized phylogeny, which is the QP1 tree. The *x*-axis represents the number of leaves in the corresponding trees. The corresponding linear regression lines are plotted as well. The Pearson correlation coefficient for both lines exceeds 0.95.

RIATA-HGT (version 2.4^46^), a program that searches for the SPR distance where an SPR operation means *Subtree Pruning and Regrafting*. This distance measures the number of such operations needed to transform one tree into another. We note that every HGT event results in an SPR operation that prunes the branch connected to the recipient of genetic material and regrafts it to the branch connected to the donor. Hence, we treat the number of SPRs computed by RIATA as an estimate of the number of HGT events that occurred during the evolution of the gene in question. Thus, this independent tool was used to uncover information regarding the intra-domain HGT rates in the following way: A hypothesized phylogeny based on the plurality quartets (the QP1 tree, see below) was used as a reference species tree. Out of the 6901 gene trees analyzed, 3392 trees had at most one archaeon and were designated “bacteria-only” trees, while 1110 trees had at most one bacterium and were designated “archaea-only” trees. Dealing only with those bacteria-only and archaea-only trees, Fig. 8 shows the number of HGTs computed by RIATA, as a function of the gene trees’ sizes. Each up-pointing red triangle (or down-pointing blue triangle) depicts the number of leaves in a single archaea-only (or bacteria-only) gene tree, and the SPR distance to the reference species tree. A strong linear correlation is found for both groups of gene trees, according to which there are on average 0.334 HGT events per species in an archaea-only tree, and 0.468 HGT events per species in a bacteria-only tree, which implies that indeed intra-bacteria HGTs are more prevalent than intra-archaea ones. The intervals for these values for a 99% confidence level are (0.325, 0.343) and (0.462, 0.474) respectively (computations done using IBM SPSS Statistics version 21, confidence level of the linear model exceeding 99.99%). The fact that these two intervals are disjoint suggests that the difference between the HGT rates within the two domains is fundamental.

### A strong tree signal nonetheless

It can be easily verified that asserting whether a collection of quartets is consistent (that is, whether a tree that satisfies them all simultaneously exists), though a generally exponential-time problem^47^, can be done efficiently when all $\left(\genfrac{}{}{0.0pt}{}{n}{4}\right)$ quartet topologies are known. We were therefore able to assert that both sets of plurality quartets, one based on the entire gene pool and the other based on the NUTs, are inconsistent. This is expected, as many plurality quartets were found to have low plurality scores. Despite the above, it is noteworthy that a large portion of those quartets support a strong tree signal signal, as the most of them have 60% plurality scores or more. In this part we examine the strength of the tree signal supported by the plurality quartets, by constructing a quartet-based phylogeny, assessing its quality, and comparing it with other hypothesized trees.

#### Quartet-based phylogenies

In this part we quantify how much the plurality quartets agree among themselves by amalgamating them into a unified phylogeny. Combining small trees into a single unifying big tree is called *supertree* and there are several approaches for this (see^28,29^ for some examples). In general, each such procedure, attempts to find a tree over the union of the taxa set that maximizes a given function related to the input trees in question. In general this operation requires exponential time (NP-hard), and heuristic approaches must be employed. We used our in-house method, the *weighted Quartet MaxCut* (wQMC) heuristic^48^ to combine the plurality quartets inferred into species tree encompassing the entire taxa set.

**Figure 9:**
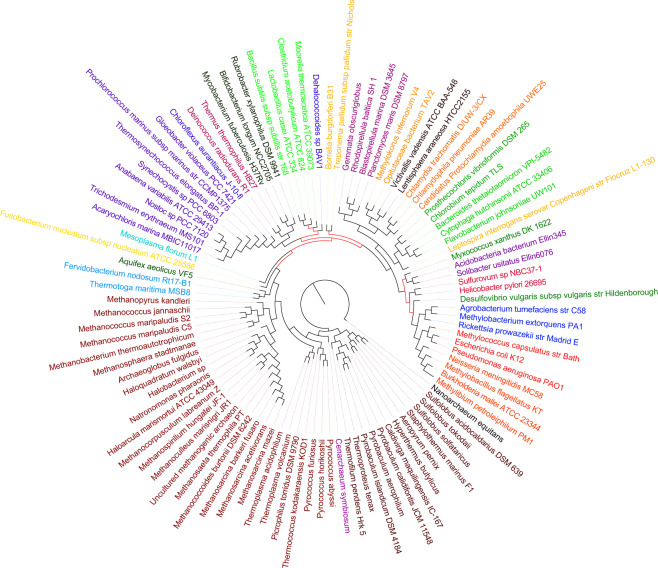
A depiction of the phylogeny, based on the 6901 gene trees pool and constructed using wQMC (the QP1 tree). The species are colored based on their phylum. Most of the branches, colored black, have high bootstrap values of 78% or more. The remaining branches, with low bootstrap values of 46–65%, are colored red, and account for the non-standard grouping of some of the species. For clarity of presentation, a rooted tree is portrayed in the figure. However, the The tree should be regarded as unrooted.

The input to wQMC, which extended the unweighted version QMC^49,50^, is a set of weighted quartet trees. Its goal is to find a tree that maximizes the total weight of the input quartets it satisfies. As two sets of plurality quartets were analyzed, two suggested phylogenies were constructed: QP1, constructed based on the entire gene pool, and QP2, constructed based on the NUTs (QP stands for “quartet plurality”). Both QP1 and QP2 satisfied roughly 90% of the plurality quartets, including all quartets whose plurality scores were at least 80%, and more than 99.99% of quartets whose plurality scores were at least 70% - a clear indication of a strong tree signal. By comparison, each one of a thousand trees that we randomly constructed did not satisfy more than 38.4% of the plurality quartets. We therefore conclude that a strong general tree trend of evolution does exist and can be extracted from the real data gene trees, even though the portion of plurality quartets that support a weak tree signal is not negligible. These results are in agreement with other studies of similar questions^3,21,22^. We remark that the QP1 tree is plotted in Fig. 9. In addition, the QP2 tree is found in S1 file, Appendix B, and both QP1 and QP2 are found, in Newick format, in S1 file, Appendices C and D. Details on how weights are assigned to the input quartets are found in Section 5.4, and additional information and statistics can be found in S1 file, Appendix A.

#### Comparison with other hypothesized phylogenies

Here we strengthen the assertion that the plurality principle can be employed to build accurate trees by contrasting QP1 and QP2 with two other reference trees on the same species set. One such reference tree was constructed in^18^ based on few of the NUTs trees taken strictly from the COG database (using a different program than ours). We denote it as the COG tree. The other reference tree was formed by concatenating several ribosomal proteins^51^ and will be denoted the ribosomal tree. First, we examined if these two trees are *a perfect phylogeny* with respect to three characters, induced by four taxonomic categories - domain, phylum, class, and order. Second, we tested the quality of the suggested phylogenies by calculating their average similarity to the input trees using two well-known similarity measures: quartet fit (Qfit) and Robinson-Foulds similarity (RF similarity). We remark in brief that a tree is a “perfect phylogeny” with respect to a given leaf partition, if the subtrees induced by each part with respect to the reference tree, are pairwise disjoint. Qfit and RF similarity are two measures, representing the number of quartets jointly agreed by the two trees and RF similarity is a tree similarity measure that expresses the number of tree splits shared by two trees, respectively. A detailed description of these measures is found in the Method section.

The results show that three of the four trees examined - QP1, QP2, and the ribosomal protein tree - are either perfect or have one misplaced leaf with respect to all four categories three classifications (Table 1). We note that when a tree is not perfect, the set of species that need to be replaced in order to make it perfect is often not unique. We therefore do not make any claims about the misplaced leaves specifically. However, the fact that no less than seven misplaced leaves exist for the COG tree in the phylum category, is testament of its relative weakness. Furthermore, with respect to Qfit and RF similarity, QP2 achieved the highest scores in all categories but one, namely: the average RF similarity score when the suggested evolutionary tree is compared to the entire gene pool (Table 2). We therefore believe that relying on the plurality quartets improves, however slightly, the accuracy of phylogenetic construction and offers a better depiction of the evolutionary histories of the species in our study.

**Table 1:** A summary of the properties of the various classifications of the proposed reference phylogenies.

	Category: domain	Category: phylum	Category: class	Category: order
QP1 tree	Tree is perfect.	Tree is not perfect	Tree is perfect.	Tree is perfect.
QP2 tree	Tree is perfect.	Tree is perfect.	Tree is perfect.	Tree is perfect.
COG tree	Tree is perfect.	Tree is not perfect. Seven leaves misplaced.	Tree is not perfect. One leaf misplaced.	Tree is not perfect. One leaf misplaced.
Ribosomal protein tree	Tree is perfect.	Tree is perfect.	Tree is perfect.	Tree is perfect.

A phylogeny is either convex (i.e., distinguish perfectly based on the relevant classification) or not, in which case the number of taxa that need to be replaced in order to make the tree perfect is indicated. Two of the trees are perfect with respect to all three classifications.

**Table 2:** A summary of the average Qfit and RF similarity scores between the four suggested phylogenies and the entire gene pool/NUTs.

	Similarity measure			
	Qfit, entire gene pool	Qfit, NUTs	RF similarity, entire gene pool	RF similarity, NUTs
QP1 tree	0.315	0.566	0.418	0.413
QP2 tree	0.315	0.568	0.420	0.422
COG tree	0.285	0.524	0.393	0.348
Ribosomal protein tree	0.313	0.549	0.421	0.420

Specifically, the first row presents, from left to right, (1) the average Qfit between QP1 and the genes in the entire gene pool, (2) the average Qfit between QP1 and the NUTs, (3) The average RF between QP1 and the genes in the entire gene pool, (4) the average RF between QP1 and the NUTs. Similarly for the other three rows. the scores relating to the QP2 tree are highest in all categories but one (namely, RF with respect to the entire gene pool), where the scores of the QP2 tree and of the ribosomal protein tree are almost identical.

## Discussion

In this paper we re-examine a set of species and gene trees already published by Puigbò *et al*.^18^. Through the development of the concept of quartet plurality distribution (QPD^32^), which enables one to combine information about quartet topologies from an aggregate of gene trees, we reveal evidence for an average low propensity for inter-domain HGTs (involving one archaeon and one bacterium), which implies the existence of an archaea-bacteria HGT barrier. Furthermore, our findings suggest that HGTs occur more frequently within the bacteria than within the archaea. Gaining information on the scarcity of inter-domain HGTs, as well as on the frequency of intra-domain HGTs, using such analytic tools, was to the best of our knowledge never reported.

One may suggest that the evidence for an HGT barrier is simply due to the specific set of species that was chosen for the assay, and if a different set of species which thrive in less restricted habitats was chosen, HGT events would have become more probable and the evidence of an HGT barrier would have all but disappeared. Indeed, the habitats of several species in this study, such as *Helicobacter pylori, Neisseria meningitidis* and *Burkholderia mallei* that live in human hosts^52–54^ or the thermophilic *Thermoplasma volcanium, Methanococcus jannaschii* and *Thermus thermophilus*^55–57^, are quite restricted. However, we note that many other factors apart from physical adjacency may influence HGT, such as toxicity^58^ (including instances where gene toxicity is manifested in the form of increased gene dosage and expression), gene function and complexity^21,59,60^, genome sequence similarity and phylogenetic proximity^61,62^, restricted recombination^63^ and lack of compatibility between the alien gene and the recipient’s tRNA pool^64^. We also note that HGT events that affect the topology of gene trees today may have occurred (and probably did occur) in the distant past. Thus, if future studies will deal with the question of how restricted habitats impact HGT, those studies will have to evaluate physical adjacency among different species in time periods spanning millions of years. This seems to be a task of baffling complexity.

As mentioned above, Puigbò *et al*.^19^ investigated a very related question of separating the evolutionary signal into its tree and net components. They defined the “tree distances” between pairs of species that were based on quartets, and used these distances as a means of separating between the archaea and the bacteria, and also between major subgroups of these domains (see Fig. 1 therein). However, it is important to note that this separation is not necessarily the result of HGT. Indeed, applying the methods of^19^ to ten simulated species trees, effectively representing ten groups of simulated gene trees that were generated with no HGTs at all, revealed some separation between the simulated archaea and bacteria, similar to the one found in^19^, in all of the ten trees that were examined (see S1 file, Appendix A). Hence, the archaea-bacteria separation induced by the methods of^19^ cannot be automatically attributed to an archaea-bacteria HGT barrier.

An additional related paper was written by Zhaxybayeva *et al*.^17^, where a quantification of horizontal gene transfers in cyanobacteria was reported. Although the ideas in that paper are related to ours, Zhaxybayeva *et al*. only dealt with cyanobacteria, hence did not report on any archaea-bacteria HGT barrier, nor did they discuss quartet plurality scores and the conclusions that can be drawn from studying them. Furthermore, though they were able to visually present and analyze support values for individual quartets (a feasible task since their paper only involved 11 genomes), it is unclear how their method can be applied to larger groups of organisms such as the one we study, where millions of quartets should be taken under consideration.

Though the inferred plurality quartets were found to be inconsistent, meaning that there is no single tree satisfying them all simultaneously, and a non-negligible portion of the plurality quartets were found to support a weak tree signal (with close to 18% of the plurality quartets having a plurality score of 40% or less), the fact that phylogenies satisfying 90% of the plurality quartets were constructed strengthens the hypothesis that a tree depicting the general trend of prokaryotic evolution exists and can (and should) be constructed, even though the effect HGT has on the evolutionary relationships between some species is too strong to be ignored. QPD induces a natural partition of the plurality quartets based on their plurality scores, which reveals that quartets with higher plurality scores are also more likely to be satisfied by the hypothesized phylogenies (see S1 file, Appendix A). This is further evidence that the strong tree signal was not reached by chance.

This position may be challenged by others. For example, Andam *et al*. report that the tree signal may be reinforced by biased HGTs^65^. It is noteworthy that in the simulations they conduct, prior evolutionary connections may be re-established to some extent once they have been damaged by random HGT events. They do not, however, demonstrate that biased HGTs can enhance existing phylogenetic proximity. In^66^, Creevey et al. report a significant tree signal only at the tips of the constructed phylogenies, while deep branch comparisons between different gene trees were no better than random. Nonetheless, as stated by the authors themselves, this may be the result of the relatively small size of taxon samples analyzed in the paper. In^67^, Thiergart *et al*. refer to the “tree of tips”, where deep speciation events are scarcely supported by individual gene trees, while they do attain high bootstrap values. However, as evidence suggests that almost all genes undergo HGT events, such discordance between gene trees and any phylogeny is expected, while the general tree-like trend of evolution may still persist.

QPD has a few potential shortcomings: the quality of any QPD graph cannot exceed that of the gene trees on which it is based, and the conclusions one draws from a QPD may be debated. Here we focused primarily on HGT, but there are several other factors that may impact the accuracy of gene trees construction in a negative way, such as gene duplication/loss, incomplete lineage sorting, or insufficient data. In^18^, where the gene trees we analyzed were constructed, the authors take steps to bypass the potentially deleterious effects of such factors.

We note that a low plurality score of a quartet alludes to a broad disagreement between gene trees. Paradoxically, such disagreement may occur with no HGTs, if the species under study are in close phylogenetic proximity and the tree signal is weak. However, using this argument in the context of this study to account for the quartets with low plurality scores would imply that intra-domain HGTs (and especially intra-bacteria HGTs) are less common than inter-domain HGTs, which is highly improbable.

QPD and the ideas pertaining to it may be relevant to additional questions. For example, refining the HGT model assumptions may facilitate a quantitative assessment of the rates of HGT in nature. Another interesting question is the validation of evolutionary theories. Indeed, our results clearly (and unsurprisingly) show that HGT events in nature are non-uniform. A related question that is still debated deals with the acquisition of genetic material from bacteria to archaea, and vice versa. It was suggested that several archaeal species evolved thanks to a large number of HGT events that occurred during a very short period of time^44,68^. However, the findings in these papers were challenged by other researchers^69,70^. Analyzing quartets comprised of 2 archaea and 2 bacteria, especially the ones that do not agree with the plurality quartets, may reveal evidence to support either side of the debate: if the hypotheses of^44,68^ regarding ancient HGT are correct, then these quartets should induce the same compartmentation on all descendant species. However, Though this is beyond the scope of this paper.

## Conclusions

In this work we discuss trends of horizontal gene transfers (HGTs) in the prokaryotic world. We present evidence suggesting the existence of a barrier against HGT between the archaea and the bacteria, making successful HGT events between the two domains relatively rare. By comparison, our findings imply that HGTs involving two archaea are moderately common and HGTs involving two bacteria are the most common. We rely mainly on the concept of quartet plurality distribution (QPD^32^), which enables us to combine information about quartet topologies from an aggregate of gene trees. We reach our conclusions through the analysis of real data QPDs and comparisons to simulations of different models of uniform and biased HGTs. In addition, RIATA-HGT is used to corroborate our results in an independent analysis. Finally, though a substantial portion of the plurality quartets derived from the gene trees in our study support a weak tree signal, we show that a strong tree-signal of evolution persists by constructing phylogenies that satisfy approximately 90% of the plurality quartets and achieve scores that are comparable to (and often better than) other hypothesized phylogenies in a number of tests.

Finally, we note that the quest for the causes for this non-uniform HGT pattern that we and others have pointed, and in particular the archaea-bacteria barrier, is intriguing and deserves independent research. Nevertheless, this question is significantly harder as it involves species and conditions that do not necessarily exist at present time.

## Methods

### Definitions

We now define several terms used in the paper. These are quite standard and can be found at e.g.^33,48^ but are presented here for completeness.

#### Phylogenetic trees

For a set of species (denoted *taxa*) ${\mathcal{X}}$, a phylogenetic ${\mathcal{X}}$-tree *t* is a tree for which there is a one to one correspondence between ${\mathcal{X}}$ and the set of leaves of *t*. The removal of an edge (or a *branch*) from the tree disconnects the tree into two subtrees and hence induces a *split* on the set of taxa. Every unrooted tree is uniquely defined by its collection of induced tree splits. The split $(U,{\mathcal{X}}\,\backslash U)$ that is identified by the edge *e* is denoted as *e*_*U*_ or ${e}_{{\mathcal{X}}\backslash U}$ alternatively. Let *t* be an ${\mathcal{X}}$-tree and ${\mathcal{A}}\subseteq {\mathcal{X}}$ be a subset of the leaves of *t*. We denote by $t{| }_{{\mathcal{A}}}$ the subtree that is *induced* by ${\mathcal{A}}$ on *t* and is obtained as follows: First, all the leaves in ${\mathcal{X}}\,\backslash {\mathcal{A}}$, as well as paths leading exclusively to them, are removed. Next all internal nodes (necessarily in paths connecting leaves from ${\mathcal{A}}$) with degree two are contracted.

#### Quartets and unrooted trees

**Figure 10:**
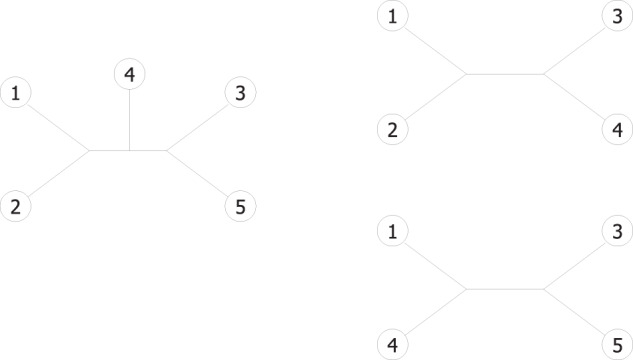
A toy example of quartets: Quartets 1, 2|3, 4 and 1, 4|3, 5 on the right are satisfied by the larger tree on the left.

A Tree *t* is *rooted* if all edges are directed away from a given node, the *root*. When edges are undirected and there are no ancestor-descendant relationships between the nodes, the tree is *unrooted*. In this work we deal with unrooted trees. In this context, the basic unit of information is a tree with four taxa, a *quartet* tree. A quartet tree with taxa {*a*, *b*, *c*, *d*} is denoted *a*, *b*|*c*, *d* if a split ({*a*, *b*}, {*c*, *d*}) is induced by one of the tree’s edges. More generally, a quartet *q* = *a*, *b*|*c*, *d* is satisfied by a tree *t* if *t* has a split separating *a*, *b* from *c*, *d* (see Fig. 10).

#### Quartet plurality score and QPD

For a given set of four taxa, say *a*, *b*, *c*, *d*, different quartets may be induced by different gene trees. For example, we assume that *a*, *b*|*c*, *d* is induced by 45% of the gene trees, *a*, *c*|*b*, *d* is induced be 30%, and *a*, *d*|*b*, *c* is induced by 25%. The *plurality quartet* is defined as the quartets that is induced the greatest number of times, and the *plurality score* of a quartet is defined as the percentage of trees inducing the plurality quartet. In our example, *a*, *b*|*c*, *d* is the plurality quartet and its plurality score is 45%. We note that unresolved quartets are discounted in this computation. When examining a collection of gene trees, and the accompanying collection of species, each 4-taxon set has its own plurality score. Thus, a collection of plurality scores may be constructed. The probability distribution of this collection of plurality scores is defined as the *Quartet plurality distribution*, or QPD.

#### Characters and perfect trees

Given a set of leaves ${\mathcal{L}}$, a *character* on ${\mathcal{L}}$ is a partition of ${\mathcal{L}}$, i.e., a division of ${\mathcal{L}}$ into disjoint subsets. Each of the subsets in a given character is called *a state*. We say that a tree *t* with leaves ${\mathcal{L}}$ is *perfect* with respect to a character *c* (equivalently, *t* *displays* *c*) if for every two states *r*_1_ and *r*_2_ in *c*, the leaves having states *r*_1_ and *r*_2_ induce two disjoint subtrees of *t*. Informally, a perfect tree is a tree that induces a perfect separation between the states of the character in question. We remark that a tree may be perfect even if all the leaves belonging to a specific state do not form a clade. A set of characters is called *compatible* if there exists a tree which displays them all simultaneously. Determining if a given collection of characters is compatible or not is called the *perfect phylogeny problem* (or *character compatibility problem*). It is, in general, NP-complete^71^.

#### Qfit

The Quartet fit tree similarity measure (^72^, *Qfit* for short) is a measure that receives two trees and computes the number of quartets shared by them, relative to the total number of induced quartets. In this paper we use a variant of this measure that was first introduced in^48^: for two trees *t*_1_, *t*_2_ we define

1 $${\rm{Qfit}}({t}_{1},{t}_{2})=\frac{2g-b}{2g+b}$$

where *g* is the number of quartets for which the two trees induce the same topology shared by the two trees, and *b* is the number of quartets for which the two trees induce different topologies that are not (more precisely, the number of 4-taxa sets that appear in both trees but with different quartet topologies) Notice that we ignore A quartet is ignored if one of its leaves is absent from one of the trees, or if its quartet topology is unresolved, i.e. a star topology. We note that according to this definition of Qfit, the mean Qfit score of two randomly chosen trees is zero (see^48^). One can apply Eq. (1) to find the Qfit similarity score between a set of quartets and a tree in an obvious way.

#### RF similarity

The Robinson-Foulds symmetric difference (^73^, *RF* for short) measures the distance between two trees by counting the number of different non-trivial splits in those trees, and dividing it by the total number of non-trivial tree splits. We were interested in tree similarity, and therefore counted the number of non-trivial splits shared by the two trees in question.

### Data and software

Real data trees were taken from^18^ for our analysis. A total of 6901 gene trees were analyzed, based on 100 species of prokaryotes (41 archaea and 59 bacteria). The full list of species is found in^18^ and is also provided in S1 table for completeness. For the simulation study, we used our own scripts to create simulated species trees and simulated gene trees. Qfit was calculated using our own script, RF similarity was calculated using Phylip^74^. Determining if a tree was perfect with respect to a given character was done using our own script. Tree reconstruction was done using wQMC^48^.

### Simulations

The underlying assumptions that dictated the generation of each gene tree in the uniform HGT simulations were: (1) the total number of HGT events impacting a given gene is a Poisson distributed random variable with parameter *λL*, where *L* is the combined length of the species tree’s branches and *λ* (defined by the user) is the “rate” of HGT events, (2) the recipients of genetic material are randomly distributed on the species tree, and (3) the donors of genetic material of each HGT event are chosen at random from the group of species contemporaneous with the recipient species. Assumptions (1) and (2) are derived from assuming that the time between two subsequent HGT events in each lineage is an exponential random variable of parameter 1/*λ* (much like speciation events along a lineage in the Yule model^75^).

In the biased HGT model, gene trees were generated such that donors and recipients of genetic material were chosen using the random HGT model with an HGT rate of 1.0 (equivalent to an average of one HGT event per one unit length on the species tree). However, an SPR operation (Subtree Pruning and Regrafting) was carried out at a lower probability, depending on the intra-domain and inter-domain HGT rates. For example, given a pair of two bacteria chosen as the recipient and donor of an HGT event, and assuming that the intra-bacteria HGT rate is 0.4, the probability of this HGT event to take place is 40%. HGT rates were divided into four categories: archaea to archaea; archaea to bacteria; bacteria to archaea; bacteria to bacteria. For intra-archaea or intra-bacteria HGTs, the HGT rate varied from 0.2 to 0.8 in increments of 0.2. For inter-domain HGTs (either from the archaea to the bacteria or vice versa), the HGT rate was either 0.1 or 0.4. Thus, a total of 64 groups of gene trees were generated for each species tree, differing in their sets of HGT rates. The simulation procedure is described in full in S1 file, Appendix A.

### Preparing the input to wQMC

As detailed in Section 2.2.1, wQMC is a supertree heuristic that receives a collection of *weighted* quartet trees as input, and amalgamates those quartets into a single unified tree. Branch lengths in the wQMC output tree are undetermined. We now elaborate more on this procedure: Let us assume we want to assign topology and weight to a given 4-taxon set {*a*, *b*, *c*, *d*}. It is easy to see that there may be three different quartet topologies based on this 4-taxaset, namely *a*, *b*|*c*, *d*, *a*, *c*|*b*, *d*, and *a*, *d*|*b*, *c*. We denote these three topologies as *q*_1_, *q*_2_, and *q*_3_ respectively. We assume that among all gene trees examined, *N*_1_ trees induce the topology *q*_1_, and similarly *N*_2_ (*N*_3_) trees induce the topology *q*_2_ (*q*_3_). If, without loss of generality, *N*_1_ ≥ *N*_2_, *N*_3_, then *a*, *b*|*c*, *d* is chosen as the quartet topology, and its weight is set to *N*_1_. This weighting scheme is justified by the theoretical work of^41^.

## Supplementary information

Supplementary Information.

Supplementary Information2.

## Data Availability

All data generated or analyzed during this study are included in this published article, and in the supporting files of the following previously published paper: P. Puigbò, Y.I. Wolf, and E.V. Koonin. Search for a ‘tree of life’ in the thicket of the phylogenetic forest. J Biol, 8(6):59, 2009. ISSN 1475-4924. 10.1186/jbiol159. http://jbiol.com/content/8/6/59.
