# Supplementary material for: A New Phylogenomic Approach For Quantifying Horizontal Gene Transfer Trends in Prokaryotes
Source: Sci Rep. 2020 Jul 24;10:12425. doi: 10.1038/s41598-020-62446-5 (PMC7381616; doi:10.1038/s41598-020-62446-5)
Supplement: Supplementary file 1 — Supplementary Information. [file 41598_2020_62446_MOESM1_ESM.pdf]

Supplementary Information for "A New Phylogenomic Approach For Quantifying Horizontal Gene Transfer Trends in Prokaryotes"

Eliran Avni[1,a] and Sagi Snir[1,b]

[1] *Dept. of Evolutionary Biology, University of Haifa, Haifa 31905, Israel*

[a] *e_avni@yahoo.com*

[b] *Corresponding author: ssagi@research.haifa.ac.il*

Appendix A: Supplementary text

This is a supplementary text to "A New Phylogenomic Approach For Quantifying Horizontal Gene Transfer Trends in Prokaryotes". We start by elaborating on the simulation procedure that was used in the paper. We then offer more details about the hypothesized phylogenies we constructed based on real data. We conclude by presenting complementary figures that were excluded from the main body of the paper.

1 The simulation procedure

We produced ten random, edge-weighted, simulated species trees over n taxa (for $n = 10, 20, \ldots 100$). Each simulated species tree was produced based on the Yule process (see [1]). Subsequently, we generated ten families of "gene" trees for each simulated species tree, each family consisting of 2500 gene trees. Each gene tree simulates a tree that is created when a species tree is subjected to a certain HGT process - a series of HGT events, consistent with a Poisson process of a constant rate. Ten different HGT rates were used, namely $\lambda = 0.1, 0.2, \ldots, 1.0$. The rate of the HGT process remained constant within each gene family, but varied from one gene family to another. The simulated species trees and gene trees were created using our own scripts. The details of how the model trees and the gene trees were created appear below.

1.1 Generating a simulated species tree

Our simulated species trees were generated based on the Yule process. The Yule process is a process in which a binary ultrametric tree is generated. Generating a Yule tree with n leaves is a process that has $n-1$ recursive stages, where in a preliminary stage, a node that represents the root of the tree is created with a time signature of zero.

In the first stage of the recursion, two exponentially distributed numbers are sampled. Let us denote them as T_1 and T_2. We choose the exponential distribution to have a parameter $\lambda = 1$, which means that for every $\varepsilon > 0$ we have $P(T_1 < \varepsilon) = P(T_2 < \varepsilon) = 1 - e^{-\varepsilon}$. After T_1 and T_2 are set, two nodes are created with time signatures T_1 and T_2. They are then connected to the root as two descendant leaves. (We can think of the time signature as representing the time of that node's creation. Thus, nodes with small time signatures are considered as "created" before nodes with large ones.) This completes the first stage. Note that once this stage is completed, there are two leaves in our tree.

Let us assume that the $(k-1)$-th stage in the recursion has been completed, resulting in a tree with k leaves. We carry out the k-th stage as follows: Two exponentially distributed numbers are sampled, let us denote them as $T_1^{(k)}$ and $T_2^{(k)}$, and two nodes are created. These two nodes are connected as descendant leaves to the leaf of the $k-1$ stage that has the smallest time signature (the leaf that was "created" first, which now becomes an inner node of the tree), and their time signatures are set at $T_1^{(k)}$ (or $T_2^{(k)}$) *plus* the time signature of their immediate ancestor. (Naturally, the time signature of each node on the Yule tree may be regarded as representing its distance from the root, or the *length* of the path from it to the root.) It is easy to see that at this point the generated tree has $k+1$ leaves. This completes the k-th stage in the recursive process.

Once $n-1$ stages have been completed and we have generated a tree with n leaves, the time signatures of all the leaves are reset to equal the smallest time signature among the leaves. This final act is what makes the generated tree ultrametric.

1.2 Generating a simulated gene tree

Based on a given species tree, we generated a set of *gene trees* by subjecting the species tree to a Poisson process of HGTs with a constant rate of events λ. This means that for each gene tree, the number of HGT events, denoted by $\#HGT$, was a random variable for which $Pr(\#HGT = m) = \frac{e^{-\lambda L}(\lambda L)^m}{m!}$ (where L denotes the total length of the species tree). Furthermore, once the value of $\#HGT$ was fixed, the m recipients of HGT events were distributed randomly and uniformly on the species tree. We remark that this is equivalent to assuming that the time between two subsequent HGT events along each lineage is an exponentially distributed random variable (like speciation events in the Yule model). An HGT event was simulated by a subtree pruning and regrafting (SPR) operation (see [4]), from the recipient of genetic material to another, randomly chosen point, at the same depth from the root in the species tree (i.e., a contemporaneous species) - the donor. When all HGTs for a gene were generated, the resulted gene tree

was ready.

2 Summary of the real data phylogeny reconstruction

As explained in the main body of the paper, we used two sets of plurality quartets, one based on the NUTs and the other based on the entire gene pool, as input for wQMC in order to construct the hypothesized phylogeny of the species in our study. The resulting trees can be found, in Newick format, in Appendices C and D below. The full list of species names and abbreviations can be found in [2] (additional data file 1). Tables 1 and 2 summarize the results of the phylogeny reconstruction. They show that both trees satisfy approximately 90% of the input quartets, and that the percentage of satisfied quartets increases with the plurality rates of those quartets.

Plurality score (%)	Satisfied quartets	Violated quartets	Total number of quartets	Percentage of satisfied quartets
33-40	419117	268505	687622	60.95%
40-50	756794	134947	891741	84.87%
50-60	314040	17025	331065	94.86%
60-70	485419	1041	486460	99.79%
70-80	761675	3	761678	100.00%
80-90	545659	0	545659	100.00%
90-100	214752	0	214752	100.00%
100	2248	0	2248	100.00%
Total	3499704	421521	3921225	89.25%

Table 1: A summary of the phylogeny reconstruction based on the entire gene pool. The reconstructed tree satisfies almost 90% of the input quartets, including almost all input quartets whose plurality rates are 70% or more.

Plurality score (%)	Satisfied quartets	Violated quartets	Total number of quartets	Percentage of satisfied quartets
33-40	347710	212826	560536	62.03%
40-50	760209	129157	889366	85.45%
50-60	306075	10789	316864	96.60%
60-70	175784	1399	177183	99.21%
70-80	331731	33	331764	100.00%
80-90	1039228	0	1039228	100.00%
90-100	587639	0	587639	100.00%
100	18645	0	18645	100.00%
Total	3567021	354204	3921225	90.97%

Table 2: A summary of the phylogeny reconstruction based on the NUTs. The reconstructed tree satisfies more than 90% of the input quartets, and almost all input quartets whose plurality rates are 70% or more.

3 Real data QPD - an extension

Similarly to what was done in the main body of the paper, we divided the plurality quartets into groups based on the number of archaea (or bacteria) they have. In the main body of the text we considered the plurality quartets that were derived from the entire gene pool, while here we consider the plurality quartets that were derived from the NUTs alone. Again, we divide the quartets into five groups (0 archaea, 4 bacteria; 1 archaea, 3 bacteria; 2 archaea, 2 bacteria; 3 archaea, 1 bacteria; 4 archaea, 0 bacteria) and show (Figure 2) how each of these groups contribute to the overall QPD of the NUTs (presented in Figure 1). We also present the independent QPDs of the five groups (Figure 3). As in the main body of the paper, in Figure 2 we see two peaks that correspond to quartets with 2 archaea and 2 bacteria and to quartets with 1 archaea and 3 bacteria, and in Figure 3 we again see that if a quartet contains one or zero archaea (three or four bacteria) then it is more likely to have a low plurality rate, compared to a quartet with three or four archaea (one or zero bacteria). These results are in agreement with what we found when we analyzed the plurality quartets of the entire gene pool, and they strengthen our conclusions regarding a high probability of bacteria-to-bacteria HGT, compared to archaea-to-archaea and to a greater extent when compared to the probability of HGTs involving both archaea and bacteria.

4 QPDs of simulated uniform HGT gene trees

Here we present the QPDs of the simulated data (Figures 4 through 13). The plots suggest that the shape of the QPD is dependent primarily on the rate of HGT events, and not on the number pf leaves in the species tree.

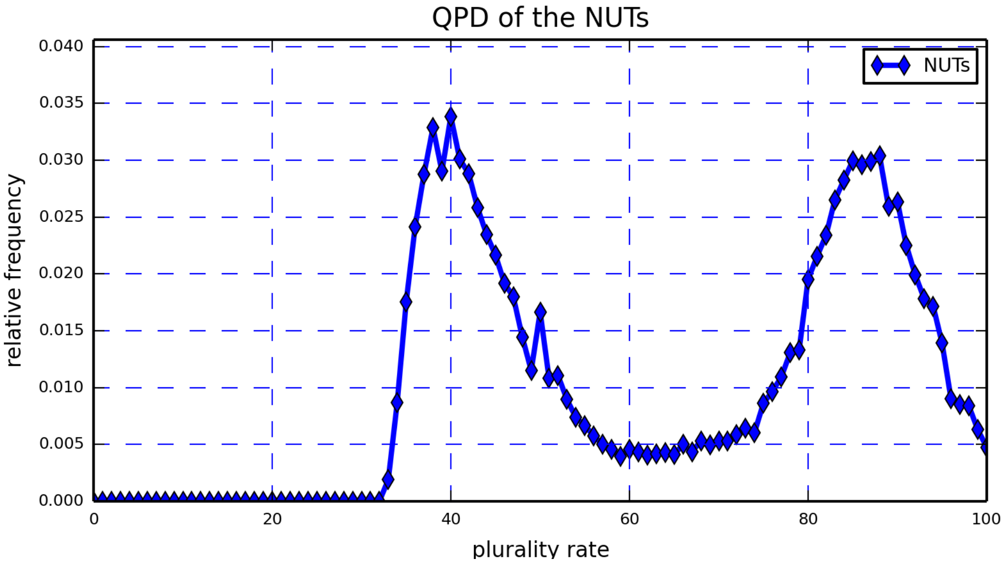

Figure 1: Real data QPD, based on the NUTs alone. We clearly see two local maxima, a phenomenon which is absent from the QPDs pertaining to the simulated data.

5 QPDs of simulated biased HGT gene trees

In an attempt to reconcile the real data QPD in the main body of the paper with our simulation process, we modified the way in which gene trees were generated. Specifically, we generated a simulated "species" tree with 100 leaves and found a node with 41 descendant leaves. That node and all of its descendants (leaves and internal nodes) were defined as "archaea". The remaining leaves and internal nodes were defined as "bacteria". HGT events were divided into four categories: archaea to archaea, archaea to bacteria, bacteria to archaea, bacteria to bacteria. Intra-domain HGTs varied from 0.2 to 0.8 in increments of 0.2, while inter-domain HGTs varied from 0.1 to 0.4. As in the uniform HGT model, recipients and donors of genetic material were chosen at random (assuming an HGT rate of 1.0, equivalent to an average of one HGT event per tree branch). However, in the biased HGT model used, the genetic transfer took place according to the relevant HGT rate. For example, in the case of a donor and recipient which were both "archaea", with an intra-archaea rate of 0.2, the genetic transfer was simulated at a probability of 20%.

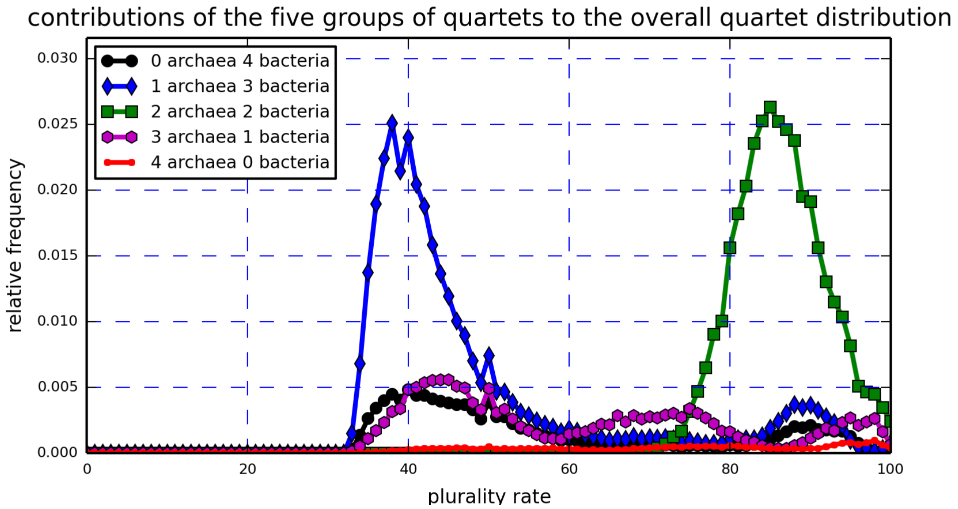

Figure 2: The contributions of five groups of quartets containing 0,1,2,3,4 archaea (equivalently, 4,3,2,1,0 bacteria) to the real data QPD based on the NUTs.

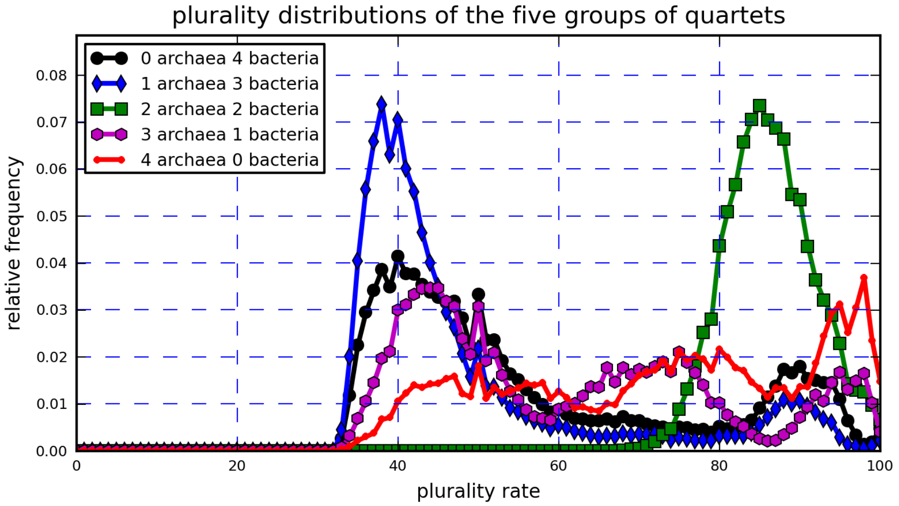

Figure 3: NUTs-based QPDs of the five groups of quartets containing 0,1,2,3,4 archaea (equivalently, 4,3,2,1,0 bacteria), plotted independently.

We present here the resulting QPD graphs thus generated pertaining to an intra-archaea rate of 0.6 (Figures 14, 17, 20, 23). Evidently, an inter-domain HGT rate of 0.1, at least twofold smaller than the smallest intra-domain HGT rate simulated, is necessary for the creation of a clear bimodal QPD graph. In addition, dealing only with the bimodal simulated QPDs, the plurality quartets were divided into five groups (0 archaea, 4 bacteria; 1 archaea, 3 bacteria; 2 archaea, 2 bacteria; 3 archaea, 1 bacteria; 4 archaea, 0 bacteria). We show, as in Figures 2 and 3, how each of these groups contribute to the overall QPD and how they compare among themselves when plotted independently. We remark that Figures 4 through 25 were plotted using randomly sampled quartet sets of size 50,000.

6 Remarks about a previously reported archaea-bacteria separation

In [3], Puigbò et al. report on an archaea-bacteria separation, derived from a "tree distance" they defined. We claim that this separation may result, at least in part, from the underlying structure of the species' phylogeny. To prove that claim, we generated ten simulated species trees with 100 leaves in each tree. As before, a node with 41 descendant leaves was identified in each species tree. This node and all of its descendants were defined as "archaea". The remaining nodes were defined as "bacteria". The pairwise distance matrices induced by each species tree were subsequently computed, using the "tree distance" defined in [3], and the resulting heatmaps were then plotted (Figures 26 through 35). This is equivalent to having no HGTs affect gene evolution at all. All heatmaps thus generated show some level of archaea-bacteria separation, which is clearly induced by the structure of the underlying phylogeny alone.

References

[1] Yule, G.U. A mathematical theory of evolution based on the conclusions of Dr. J.C. Willis, F.R.S.. *Philos Trans R Soc Lond B Biol Sci.* **213**, 21–87 (1924).

[2] Puigbò, P., Wolf, Y.I. & Koonin, E.V. Search for a 'tree of life' in the thicket of the phylogenetic forest. *J Biol.* **8**(6), 59 (2009).

[3] Puigbò, P., Wolf, Y.I. & Koonin, E.V. The tree and net components of prokaryote evolution. *Genome Biol Evol.* **2**, 745–756 (2010).

[4] Semple, C. & Steel, M.A. *Phylogenetics* (Oxford University Press, 2003).

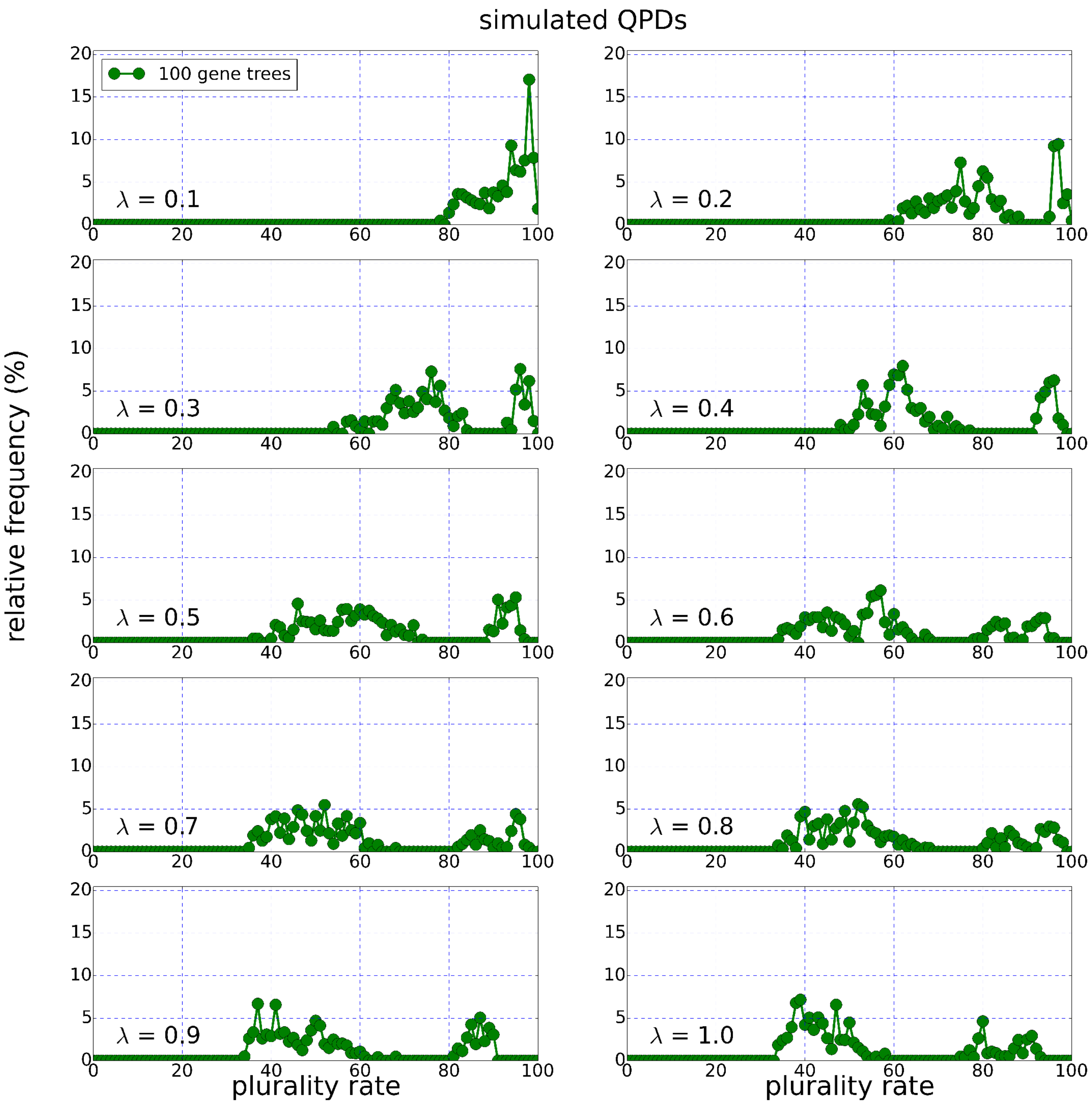

Figure 4: QPD graphs of the simulated data for a simulated species tree with $n = 10$ leaves.

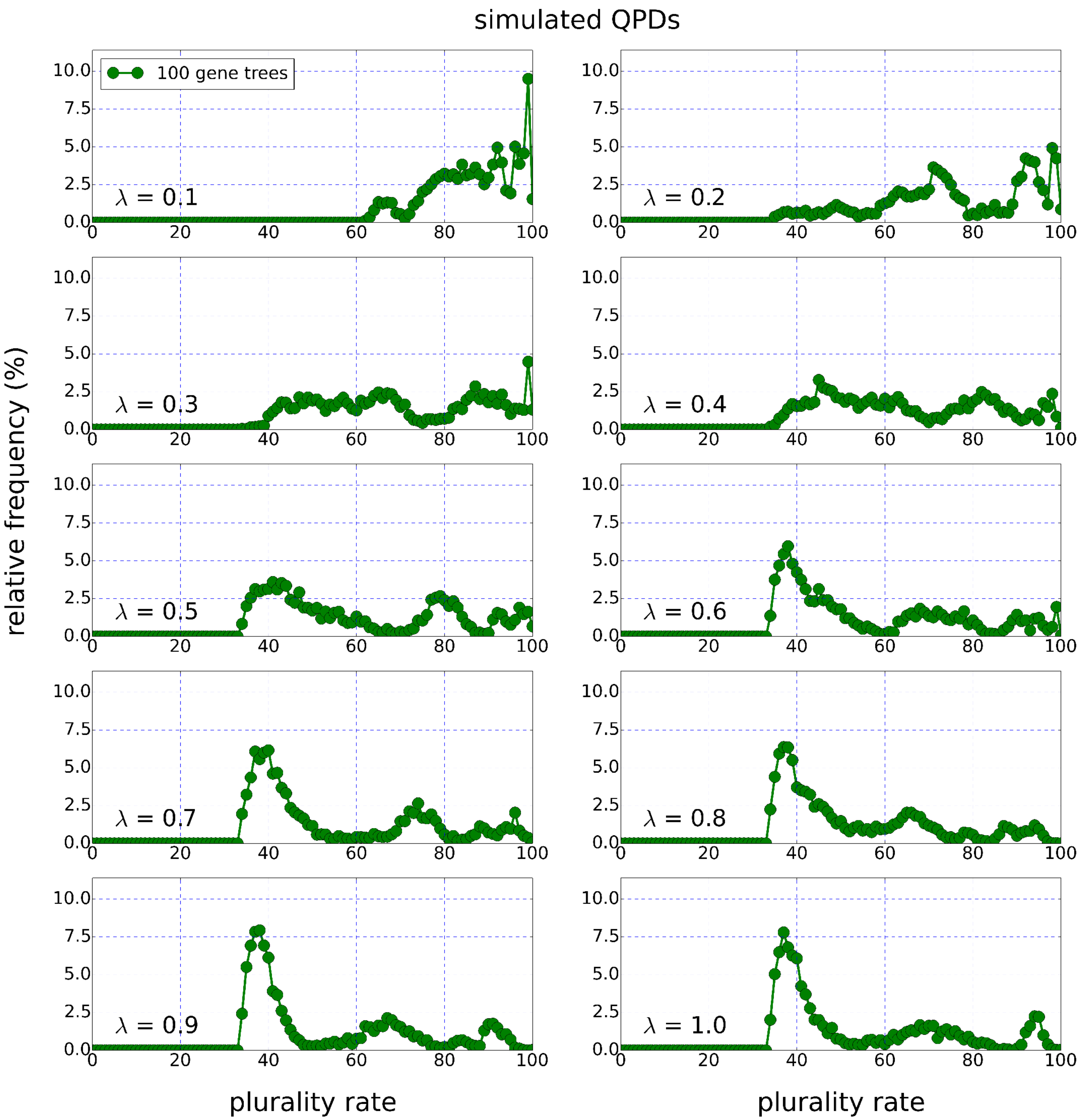

Figure 5: QPD graphs of the simulated data for a simulated species tree with $n = 20$ leaves.

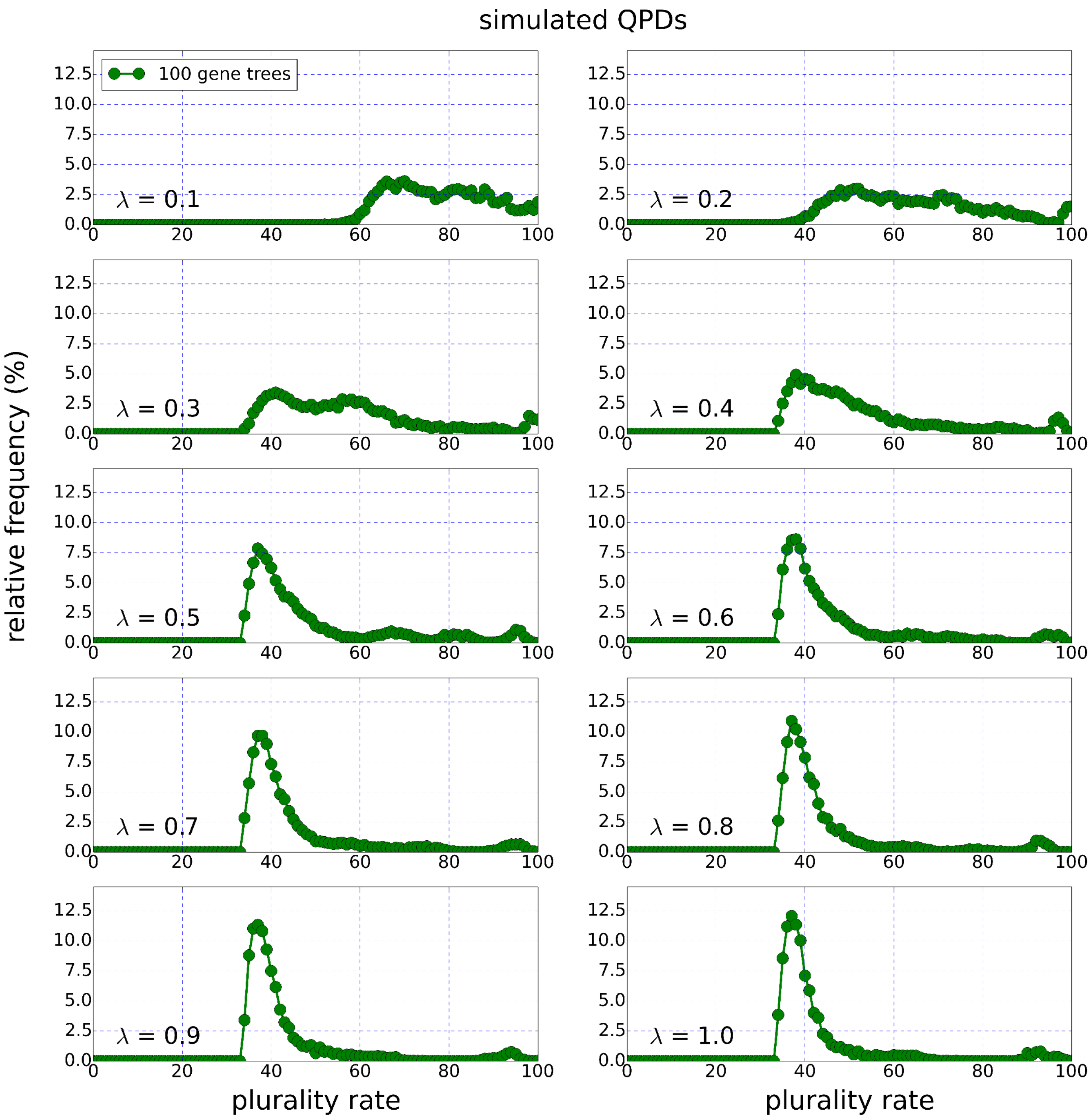

Figure 6: QPD graphs of the simulated data for a simulated species tree with $n = 30$ leaves.

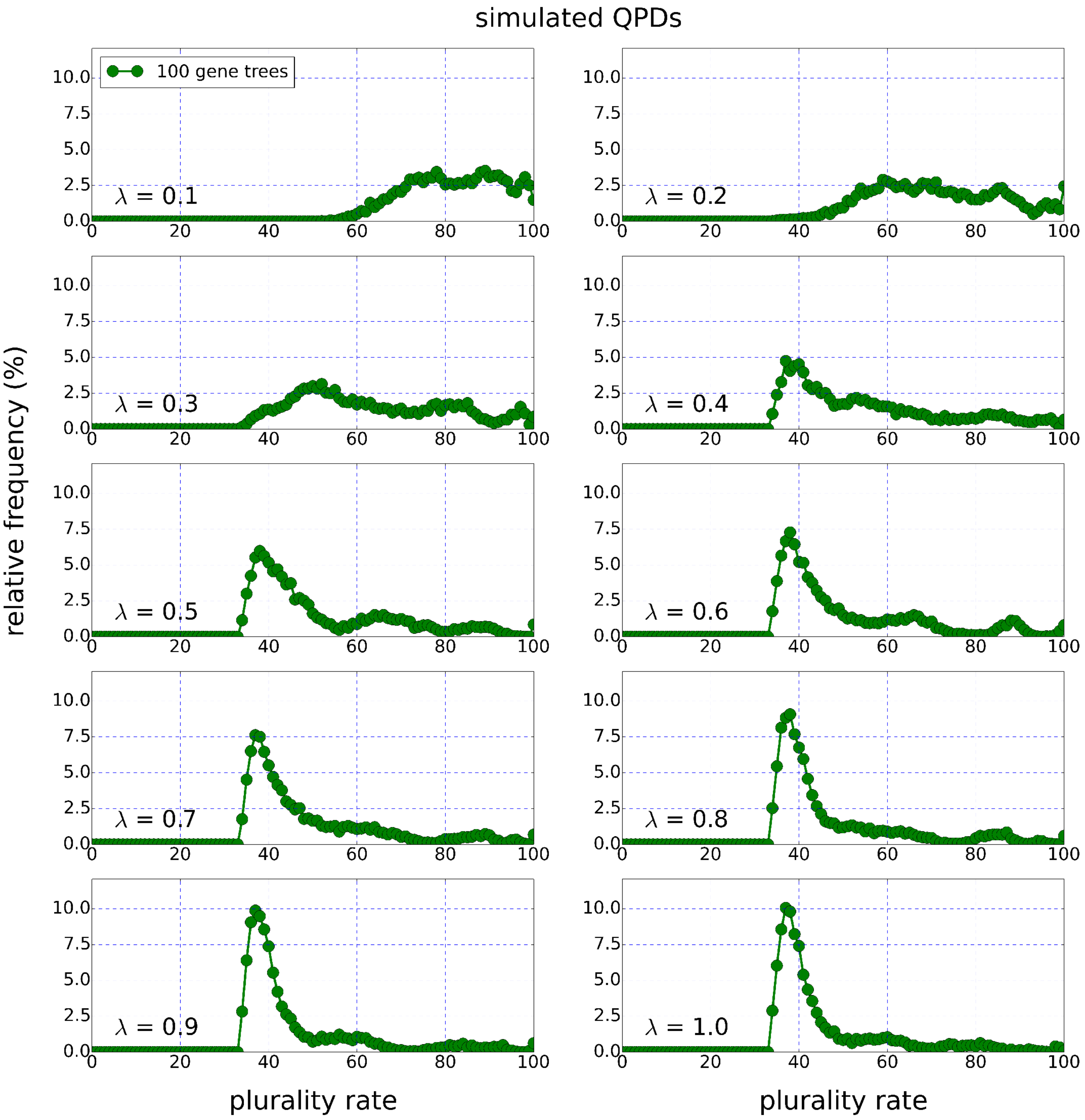

Figure 7: QPD graphs of the simulated data for a simulated species tree with $n = 40$ leaves.

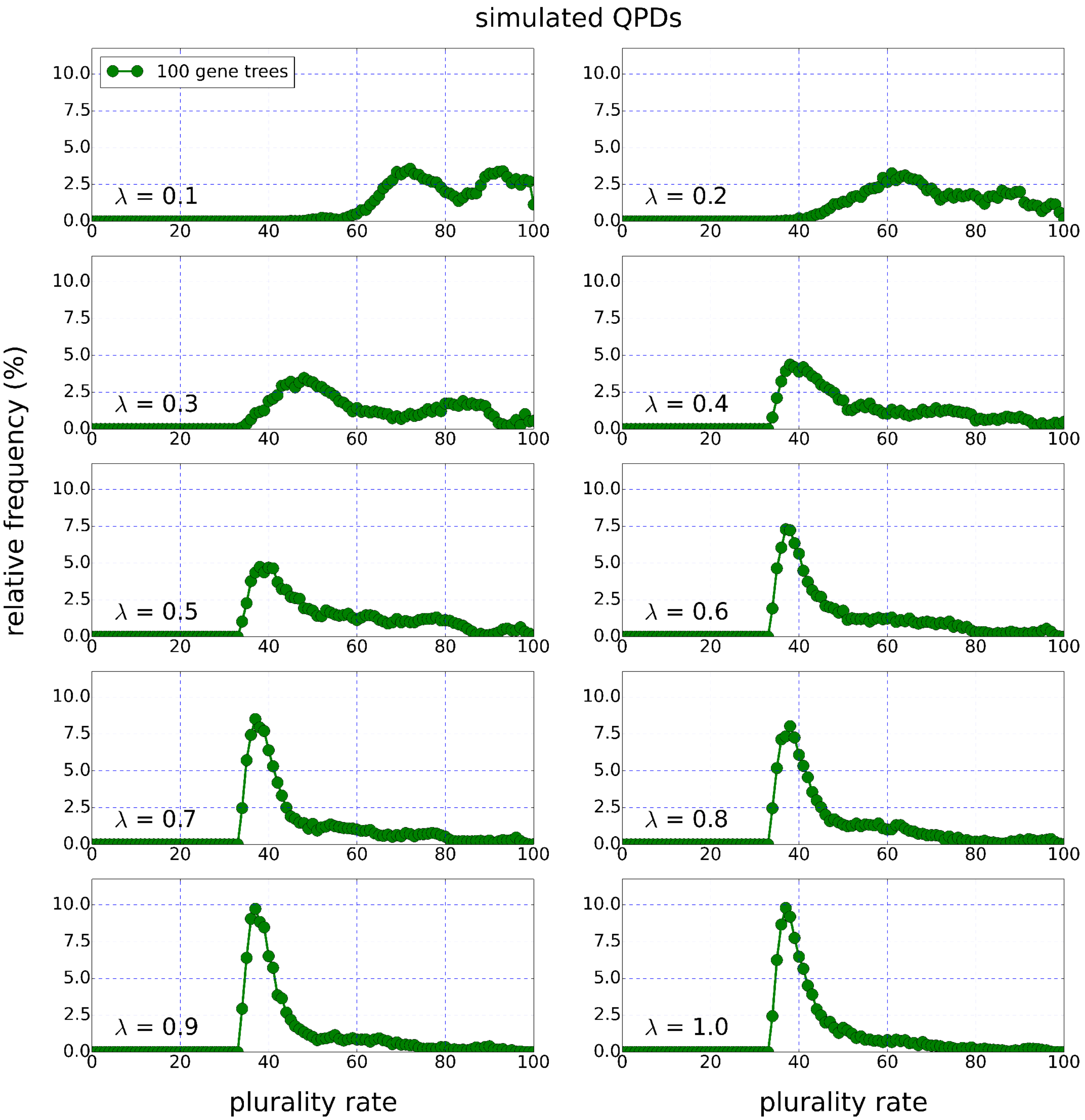

Figure 8: QPD graphs of the simulated data for a simulated species tree with $n = 50$ leaves.

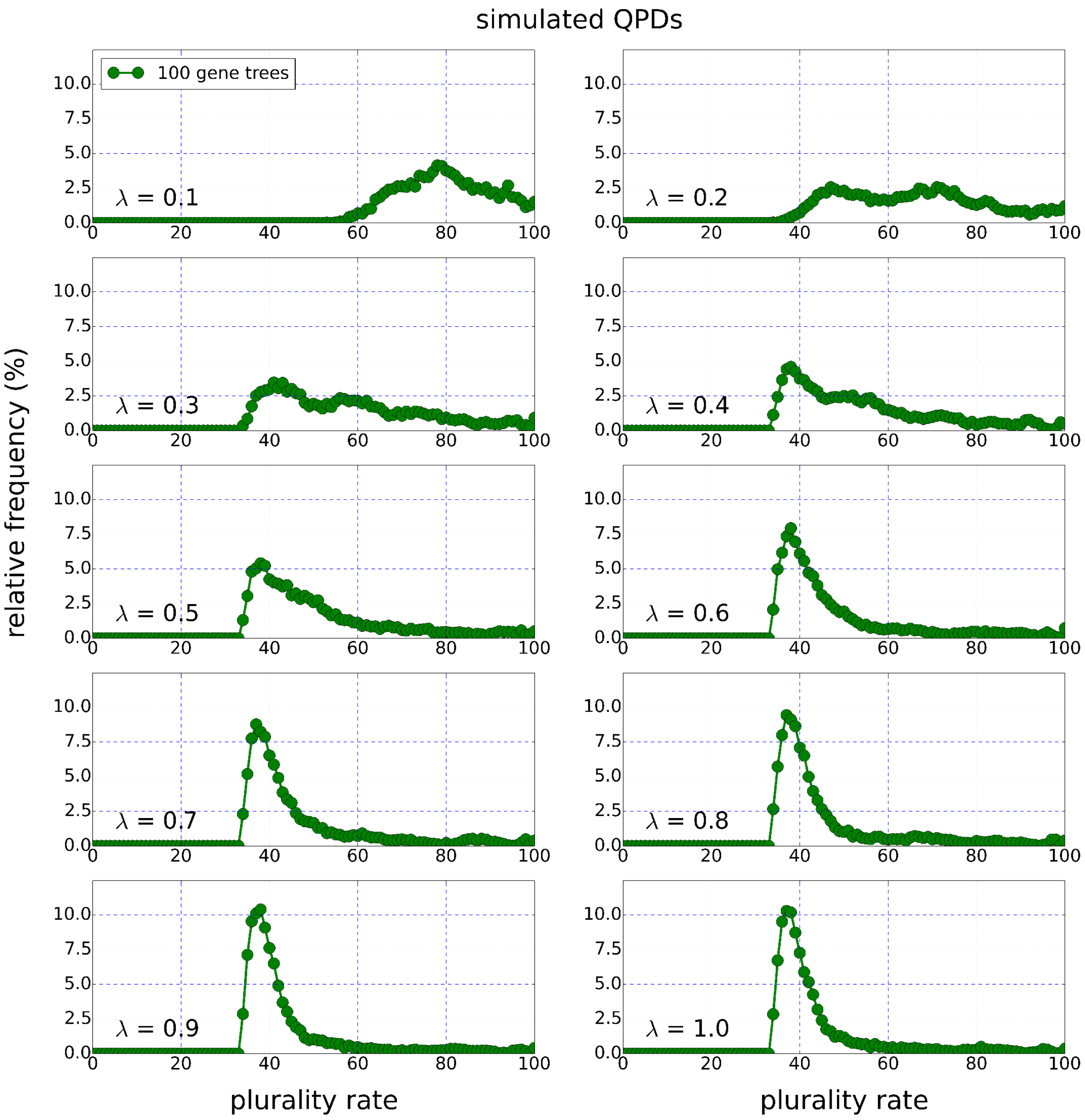

Figure 9: QPD graphs of the simulated data for a simulated species tree with $n = 60$ leaves.

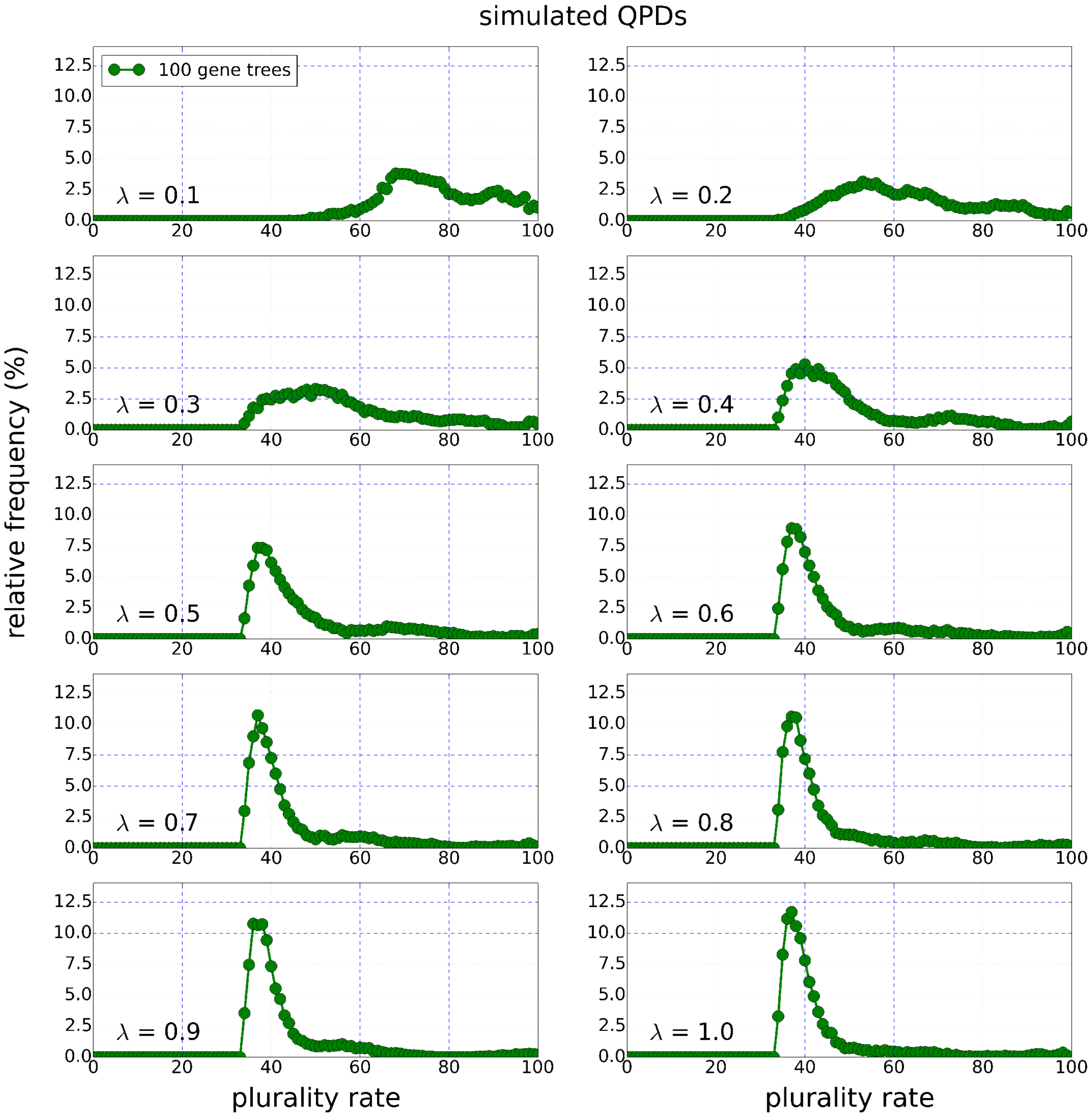

Figure 10: QPD graphs of the simulated data for a simulated species tree with $n = 70$ leaves.

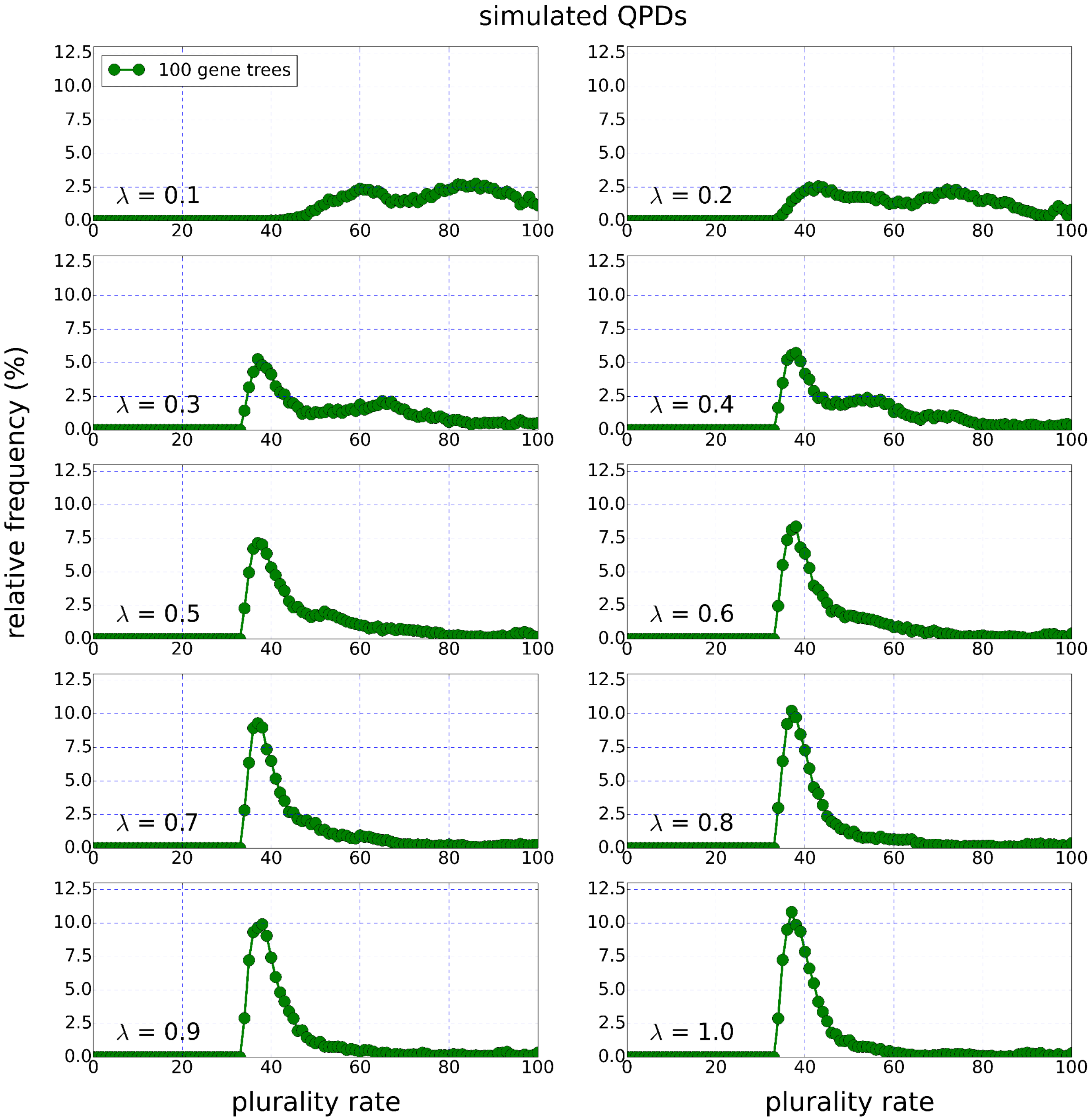

Figure 11: QPD graphs of the simulated data for a simulated species tree with $n = 80$ leaves.

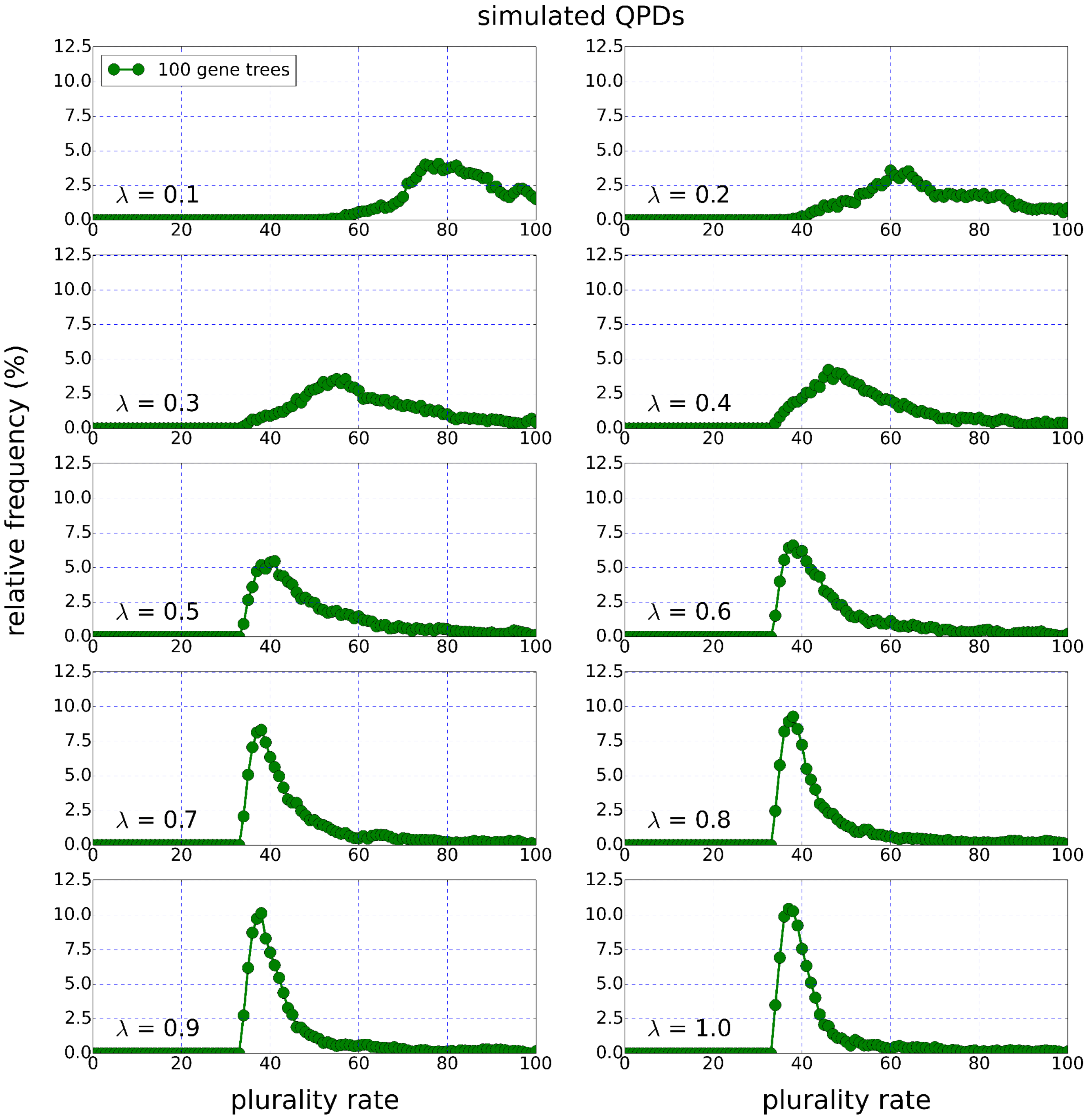

Figure 12: QPD graphs of the simulated data for a simulated species tree with $n = 90$ leaves.

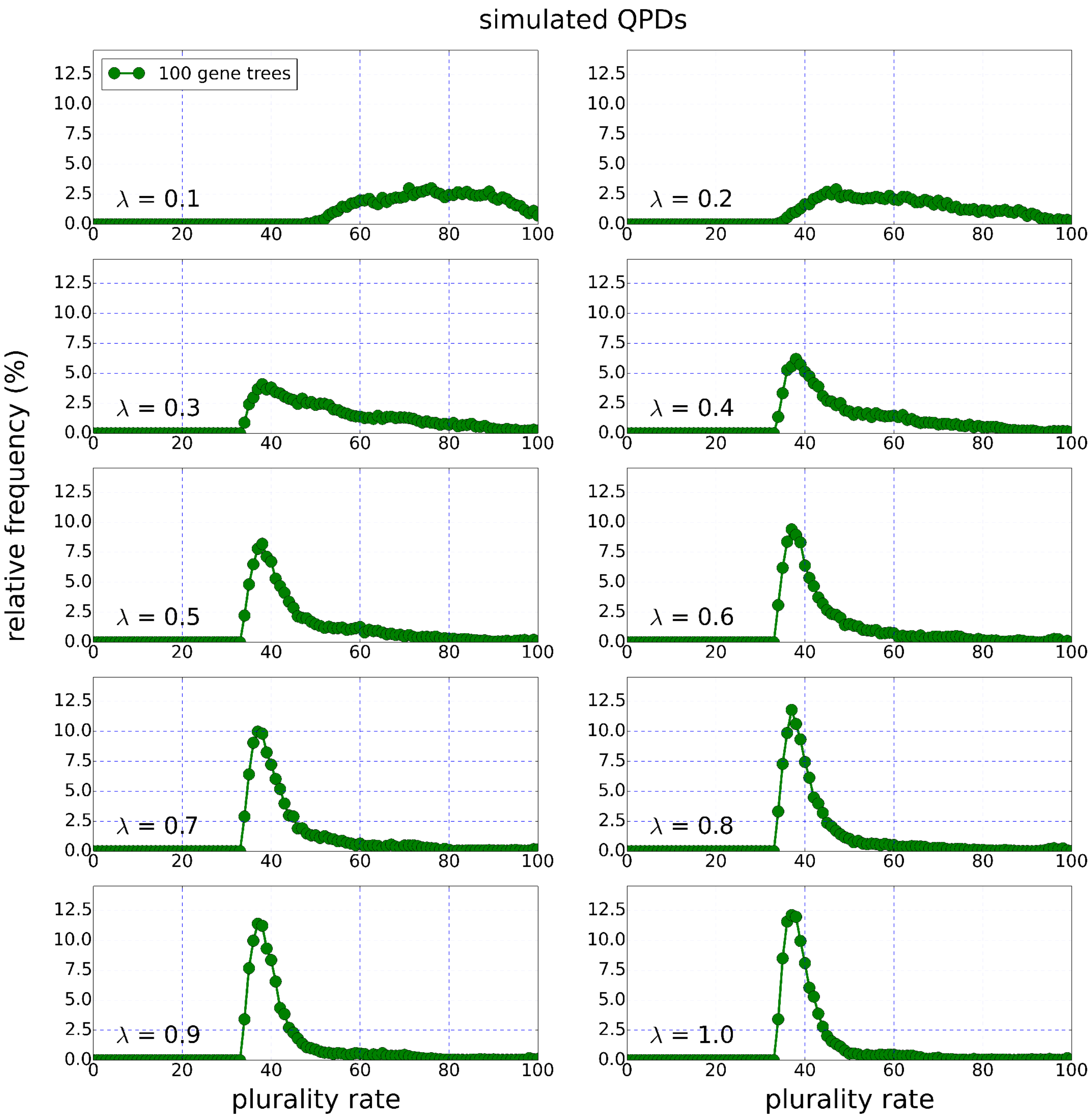

Figure 13: QPD graphs of the simulated data for a simulated species tree with $n = 100$ leaves.

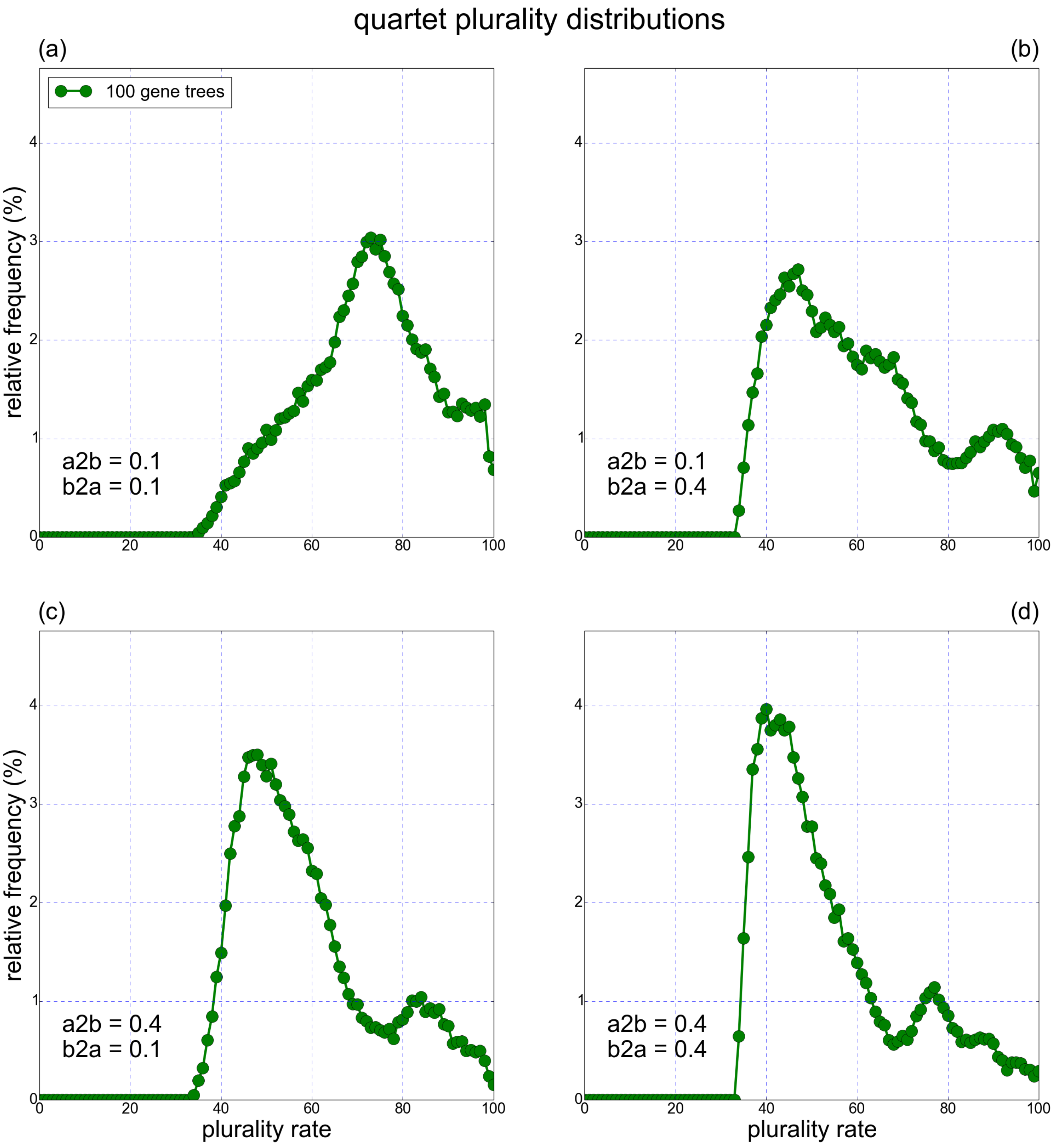

Figure 14: QPD graphs of the simulated biased HGT based on a simulated species tree with $n = 100$ leaves, with intra-archaea rate of 0.6 and intra-bacteria rate of 0.2.

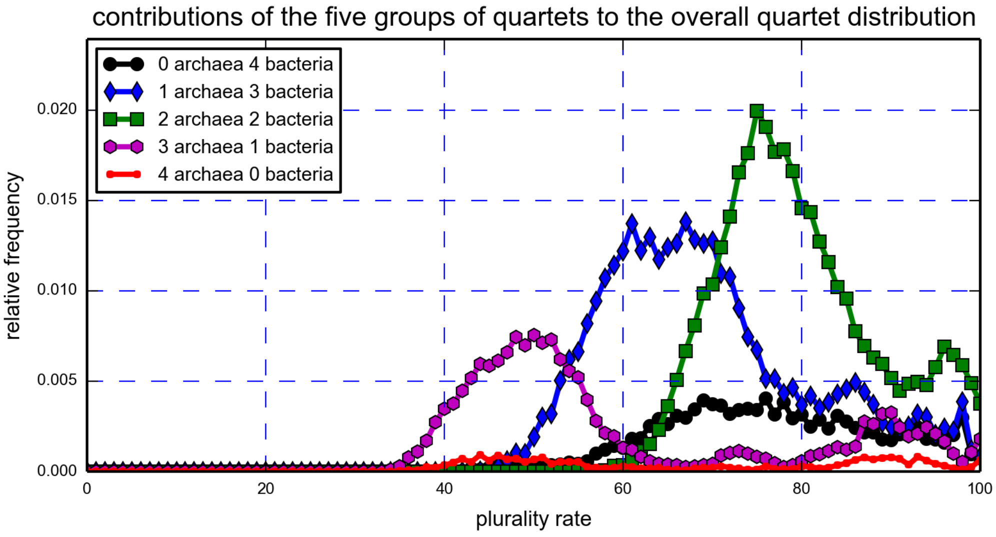

Figure 15: The contributions of five groups of quartets containing 0,1,2,3,4 archaea (equivalently, 4,3,2,1,0 bacteria) to the real data QPD based on Figure 14(a).

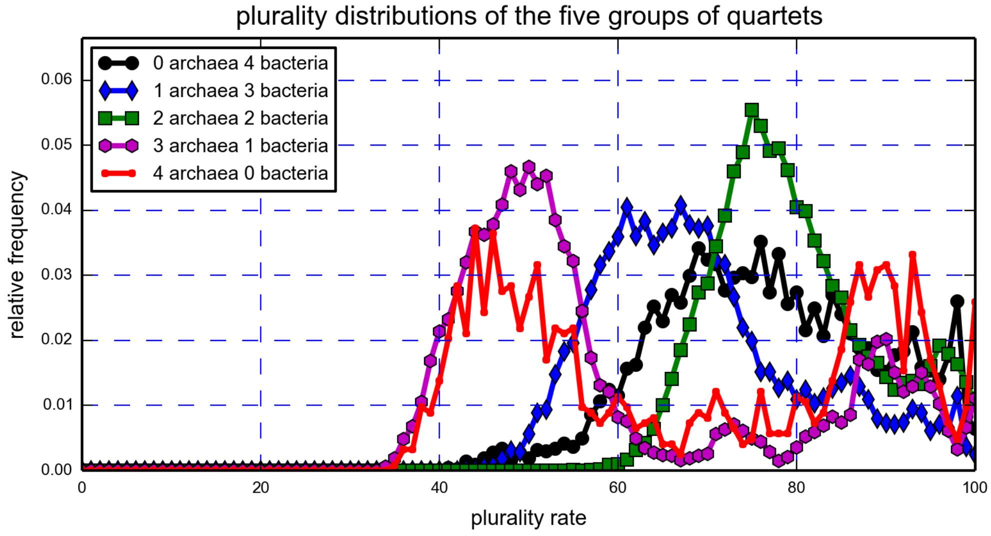

Figure 16: The QPDs of the five groups of quartets containing 0,1,2,3,4 archaea (equivalently, 4,3,2,1,0 bacteria), plotted independently based on Figure 14(a).

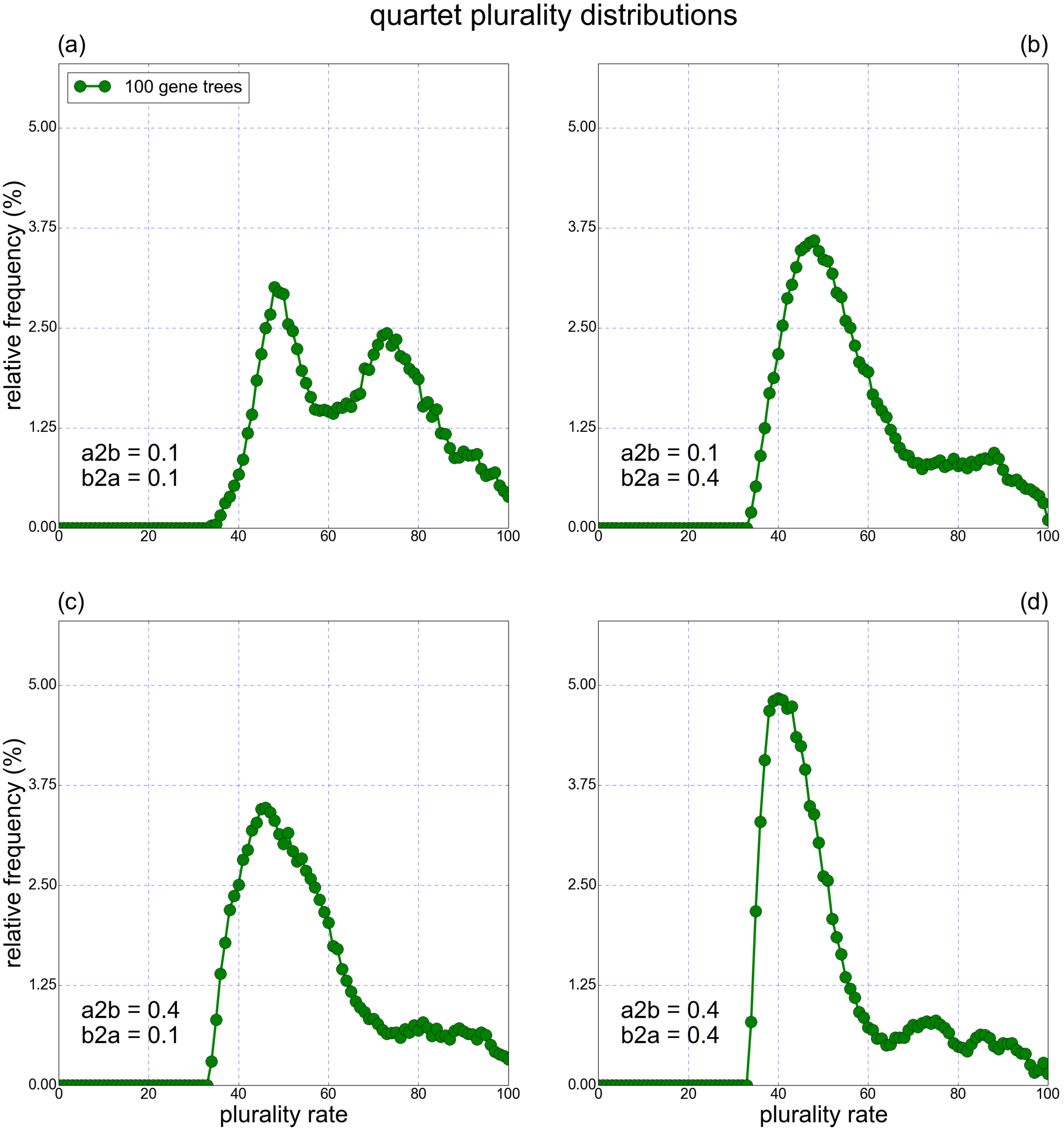

Figure 17: QPD graphs of the simulated biased HGT based on a simulated species tree with $n = 100$ leaves, with intra-archaea rate of 0.6 and intra-bacteria rate of 0.4.

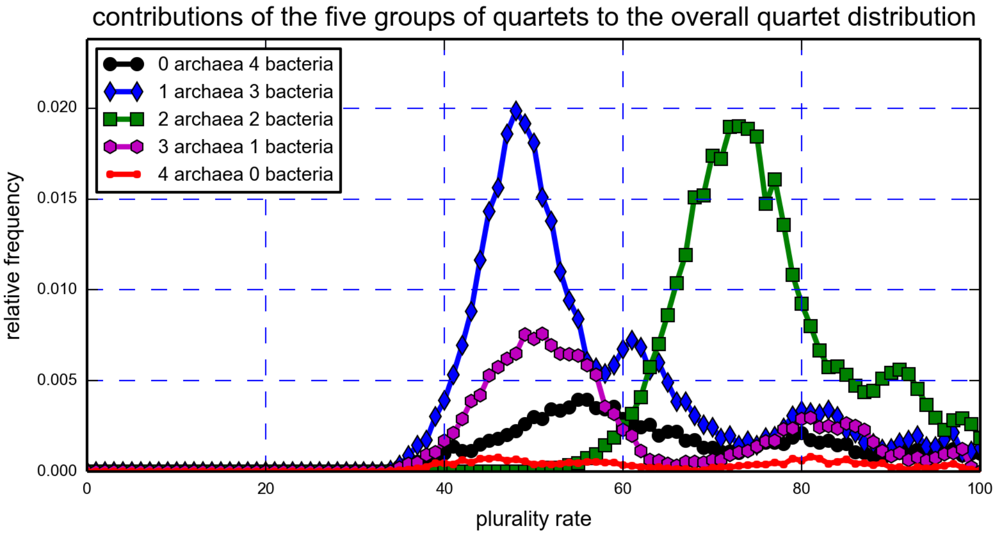

Figure 18: The contributions of five groups of quartets containing 0,1,2,3,4 archaea (equivalently, 4,3,2,1,0 bacteria) to the real data QPD based on Figure 17(a).

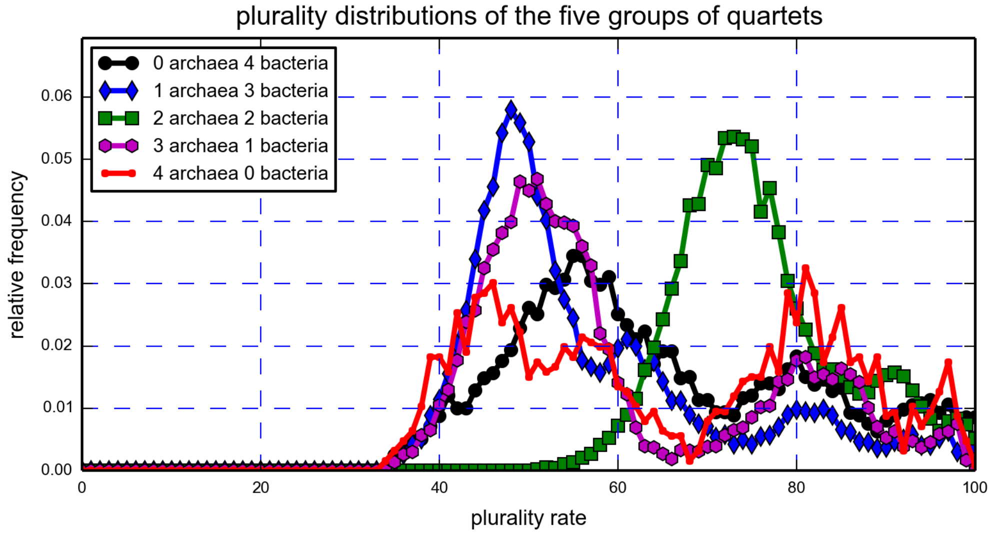

Figure 19: The QPDs of the five groups of quartets containing 0,1,2,3,4 archaea (equivalently, 4,3,2,1,0 bacteria), plotted independently based on Figure 17(a).

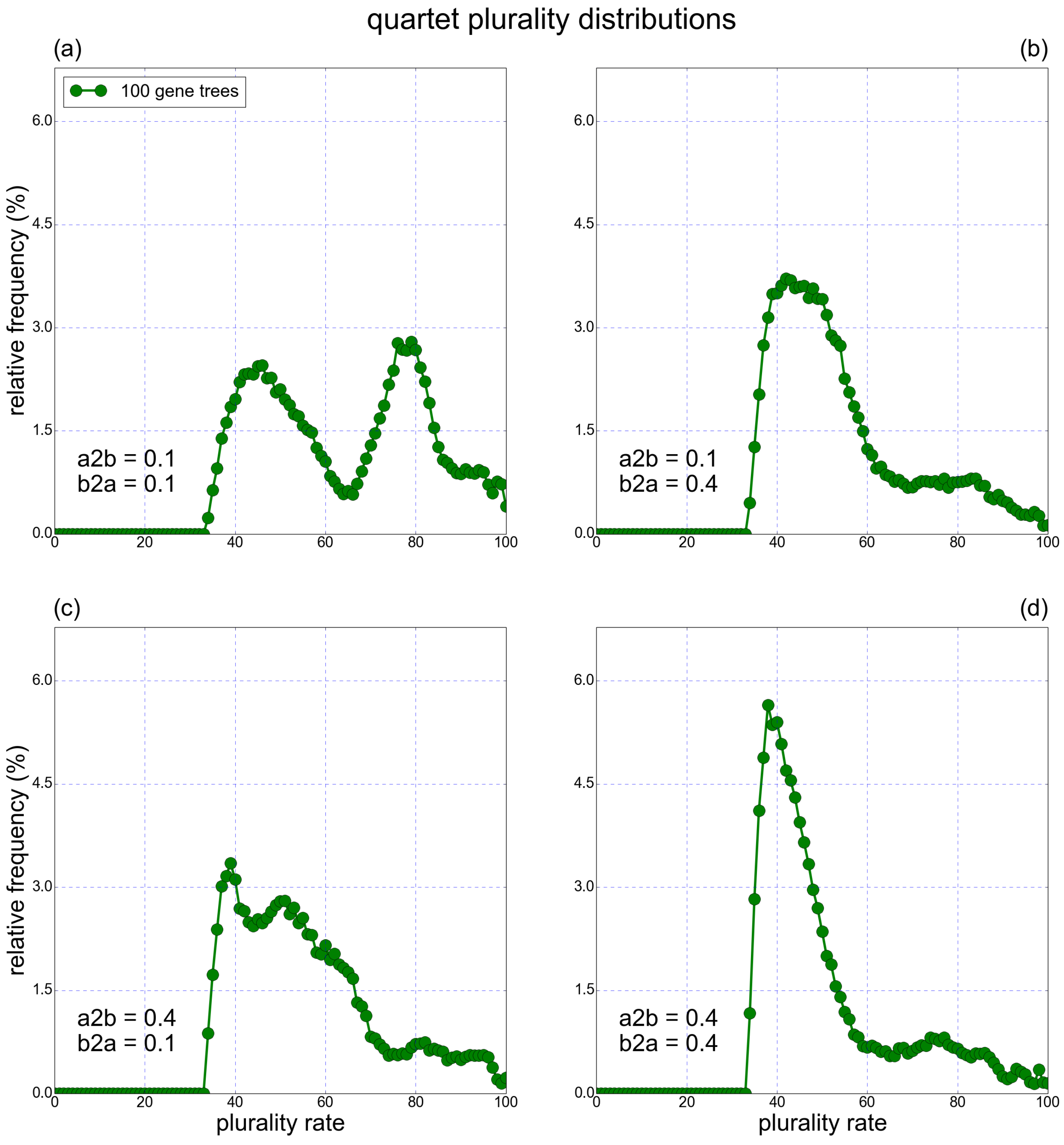

Figure 20: QPD graphs of the simulated biased HGT based on a simulated species tree with $n = 100$ leaves, with intra-archaea rate of 0.6 and intra-bacteria rate of 0.6.

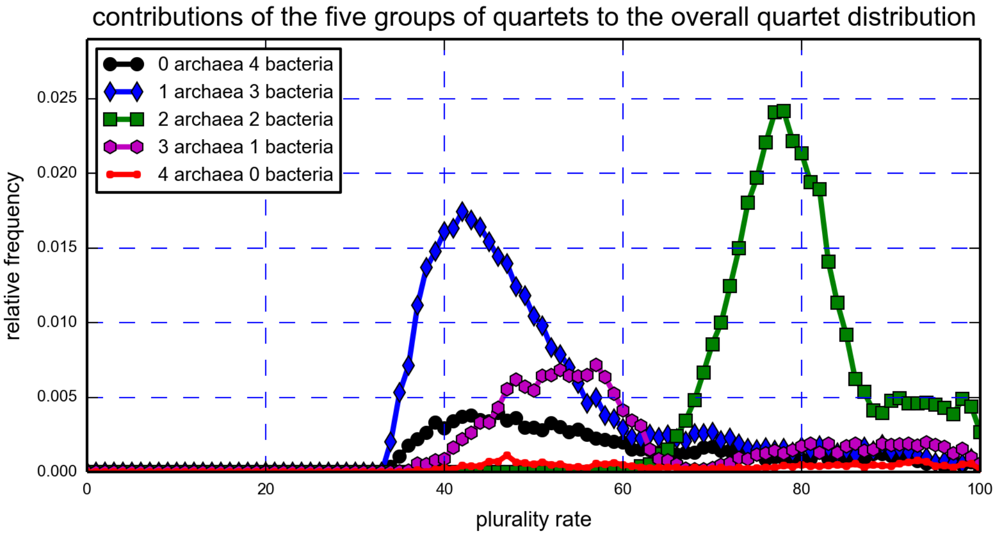

Figure 21: The contributions of five groups of quartets containing 0,1,2,3,4 archaea (equivalently, 4,3,2,1,0 bacteria) to the real data QPD based on Figure 20(a).

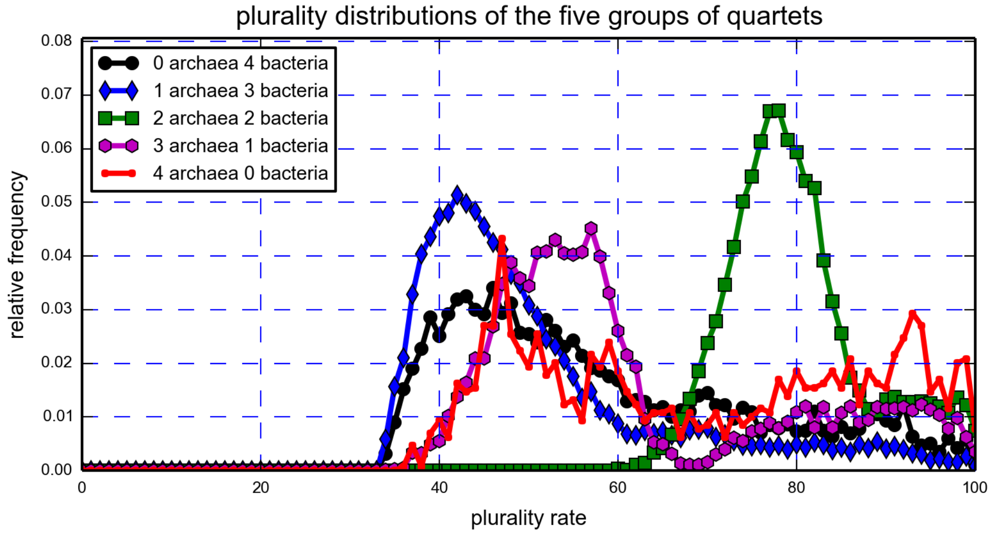

Figure 22: The QPDs of the five groups of quartets containing 0,1,2,3,4 archaea (equivalently, 4,3,2,1,0 bacteria), plotted independently based on Figure 20(a).

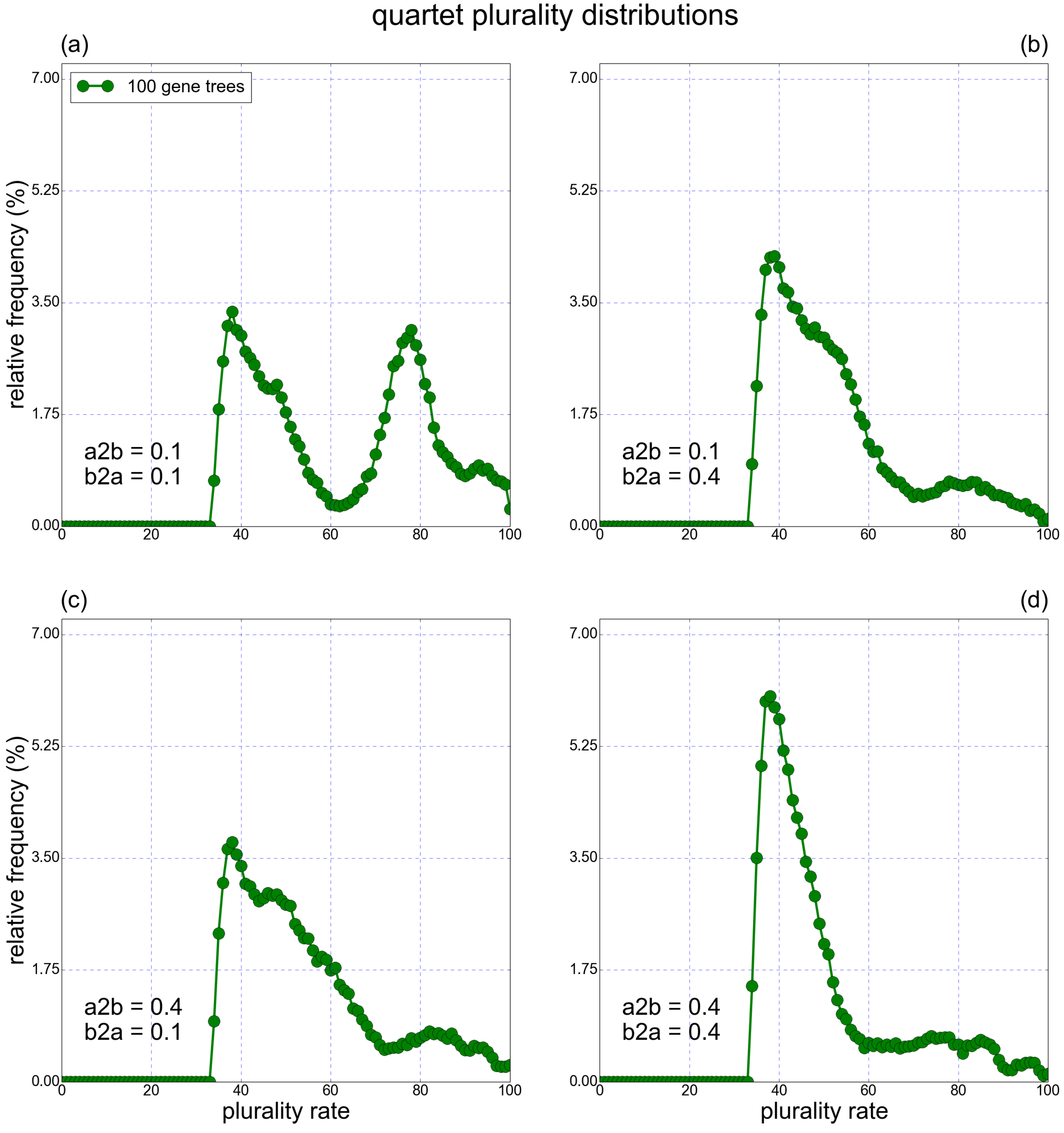

Figure 23: QPD graphs of the simulated biased HGT based on a simulated species tree with $n = 100$ leaves, with intra-archaea rate of 0.6 and intra-bacteria rate of 0.8.

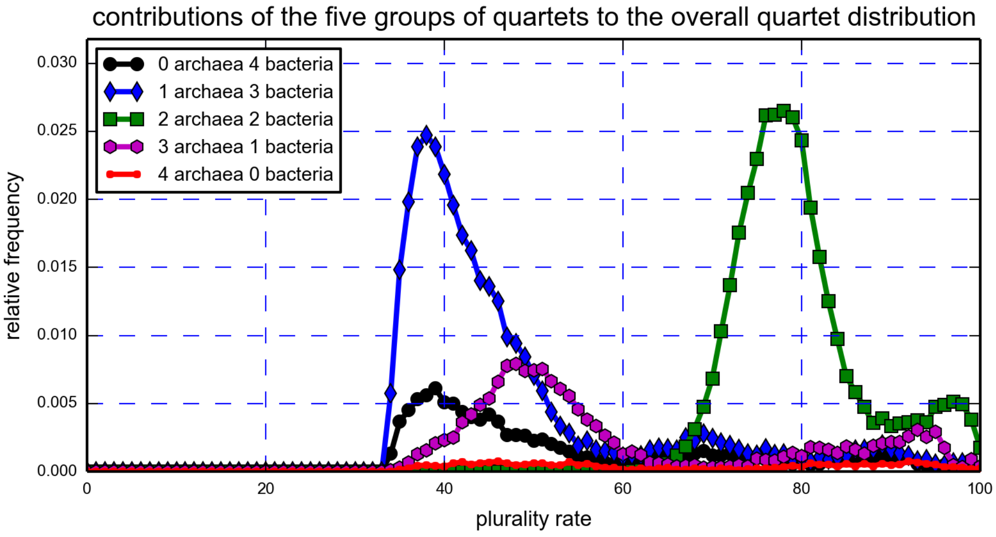

Figure 24: The contributions of five groups of quartets containing 0,1,2,3,4 archaea (equivalently, 4,3,2,1,0 bacteria) to the real data QPD based on Figure 23(a).

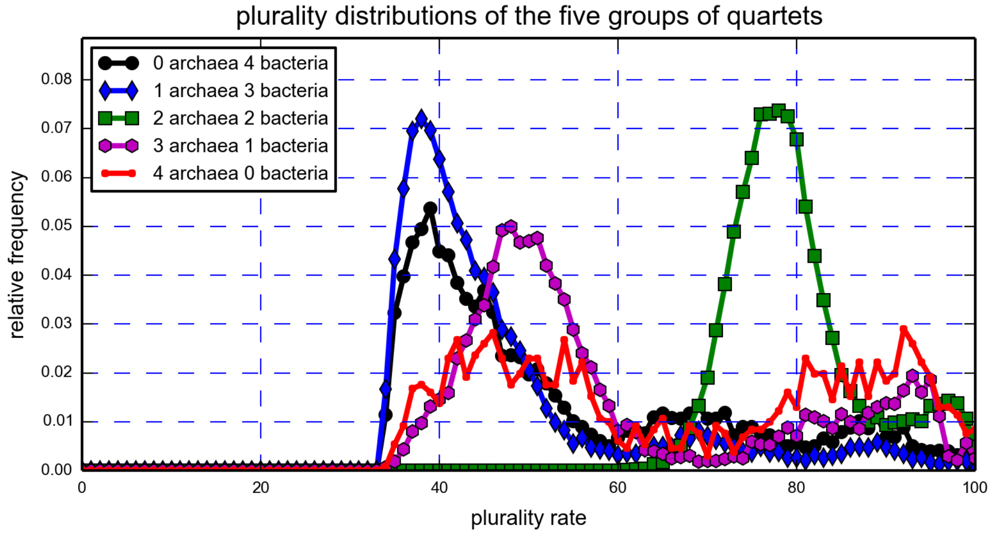

Figure 25: The QPDs of the five groups of quartets containing 0,1,2,3,4 archaea (equivalently, 4,3,2,1,0 bacteria), plotted independently based on Figure 23(a).

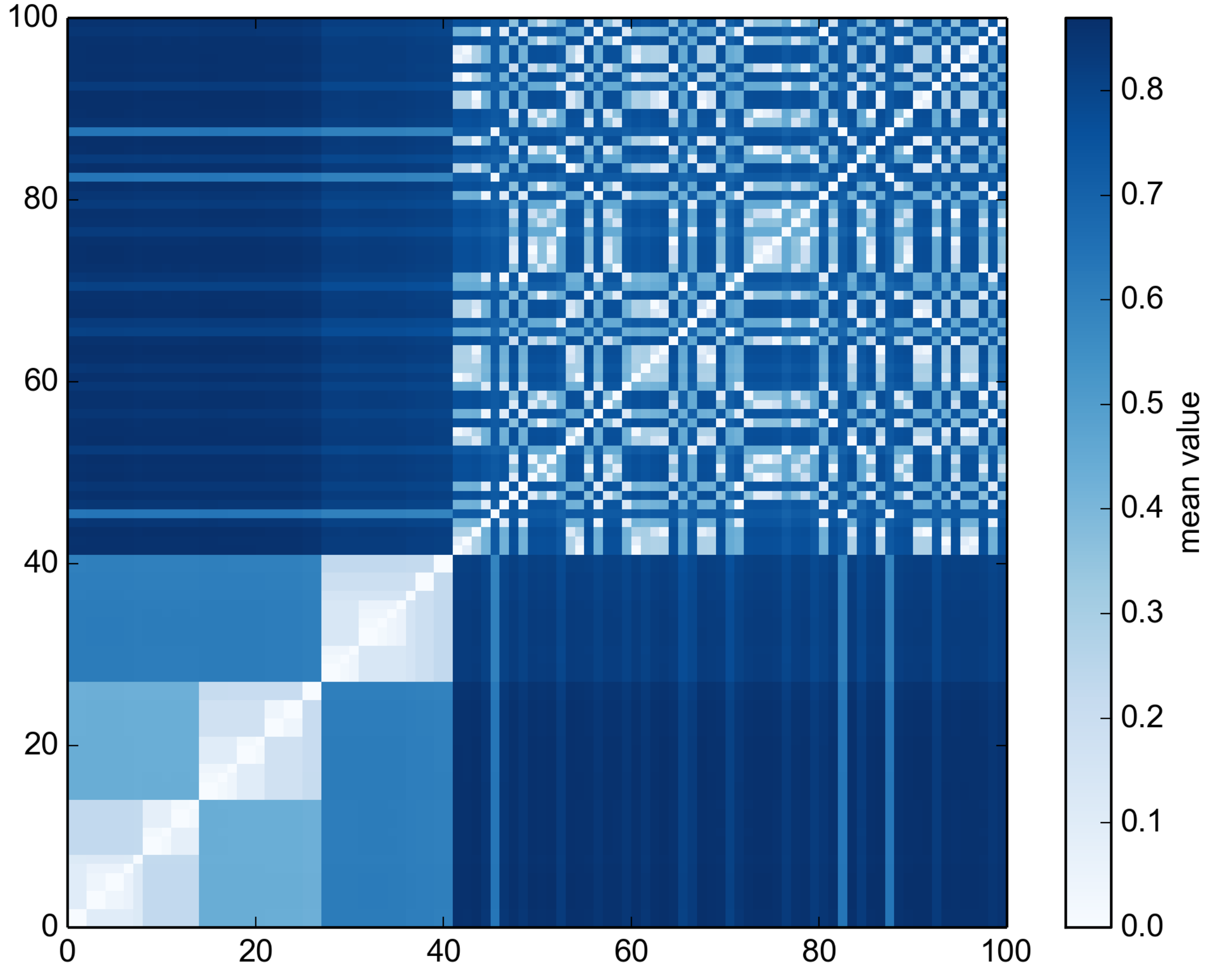

Figure 26: Heatmap of the pairwise "tree distance" matrix based on simulated species tree No. 1. Species 1-41 are "archaea", species 42-100 are "bacteria".

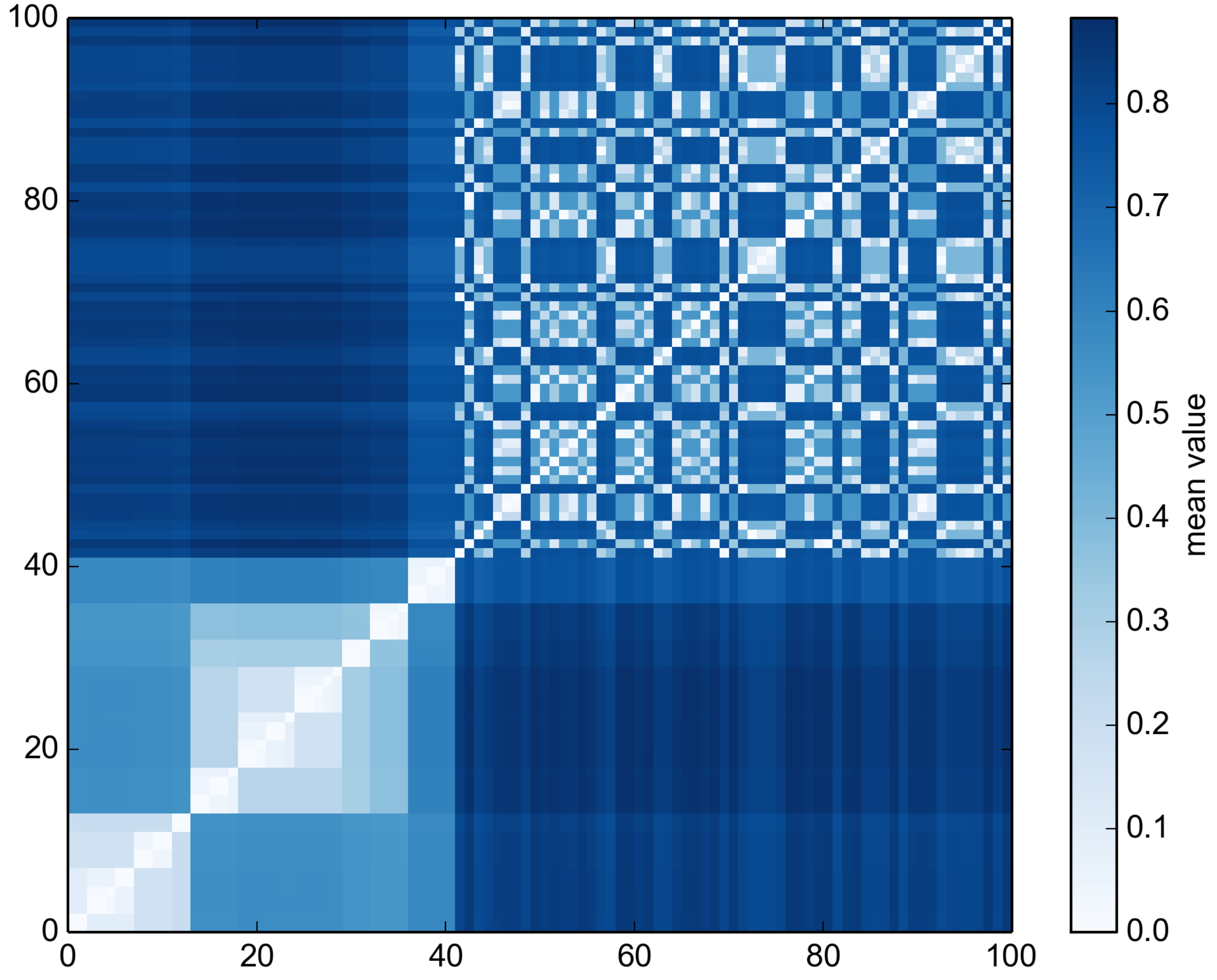

Figure 27: Heatmap of the pairwise “tree distance” matrix based on simulated species tree No. 2. Species 1-41 are “archaea”, species 42-100 are “bacteria”.

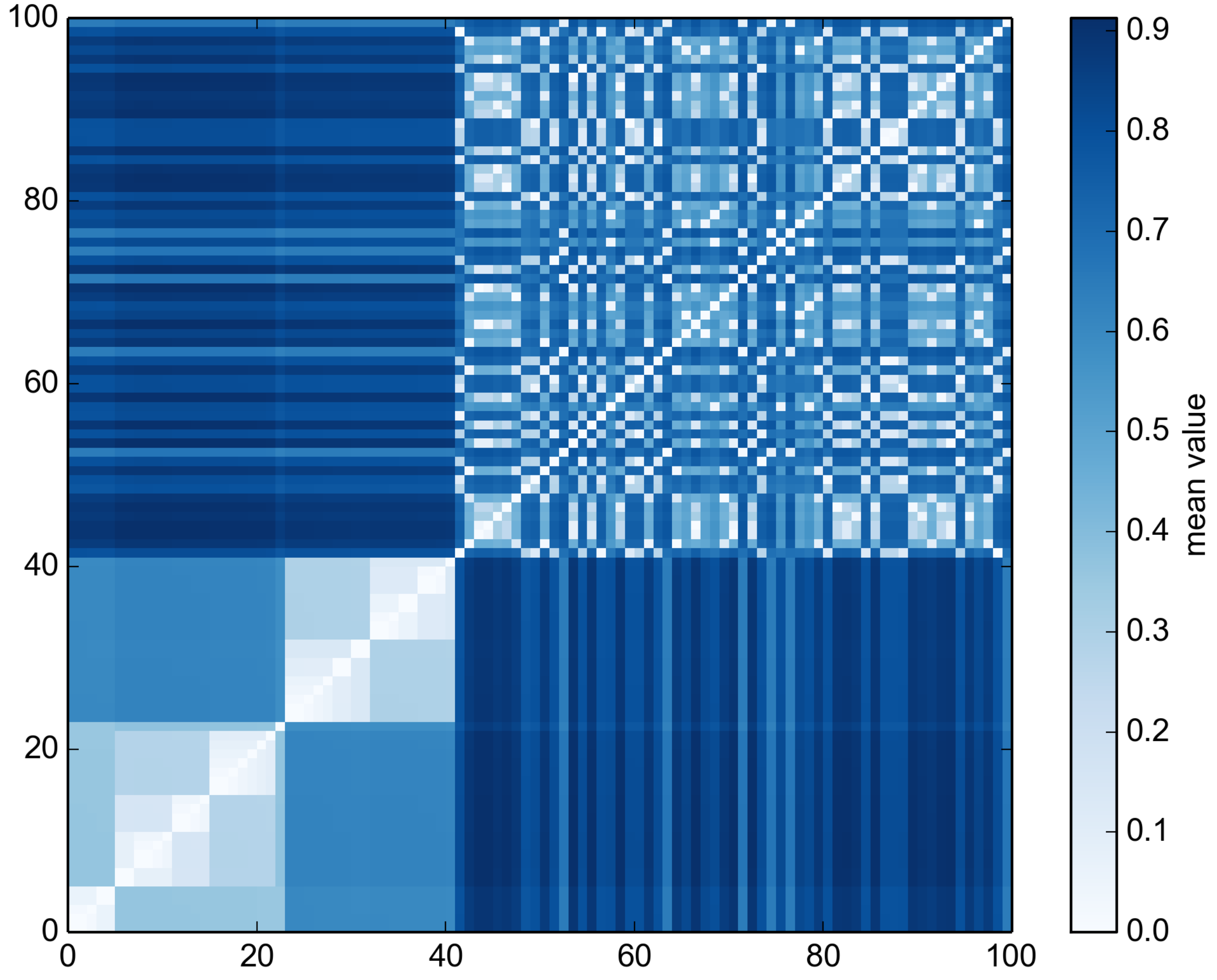

Figure 28: Heatmap of the pairwise "tree distance" matrix based on simulated species tree No. 3. Species 1-41 are "archaea", species 42-100 are "bacteria".

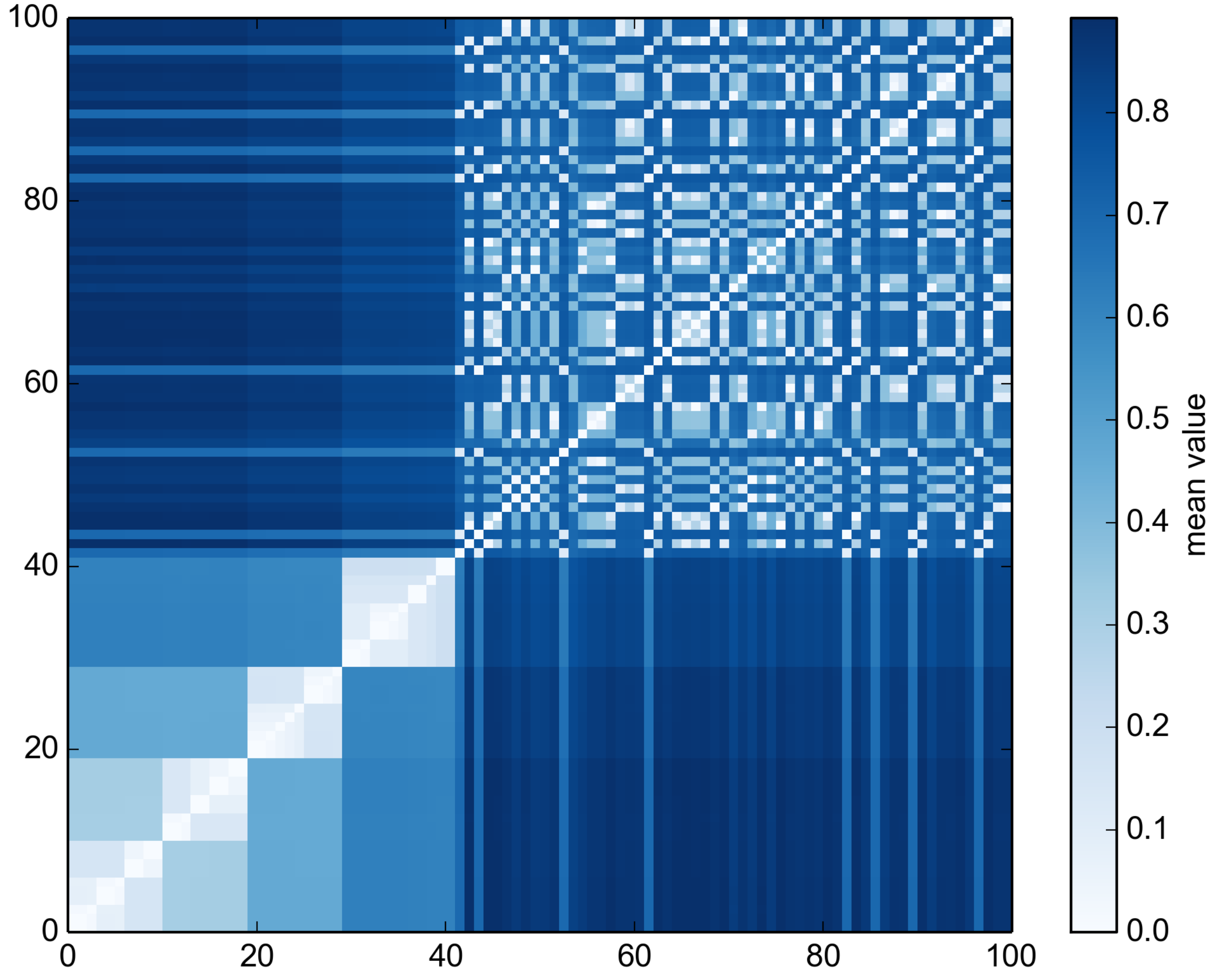

Figure 29: Heatmap of the pairwise "tree distance" matrix based on simulated species tree No. 4. Species 1-41 are "archaea", species 42-100 are "bacteria".

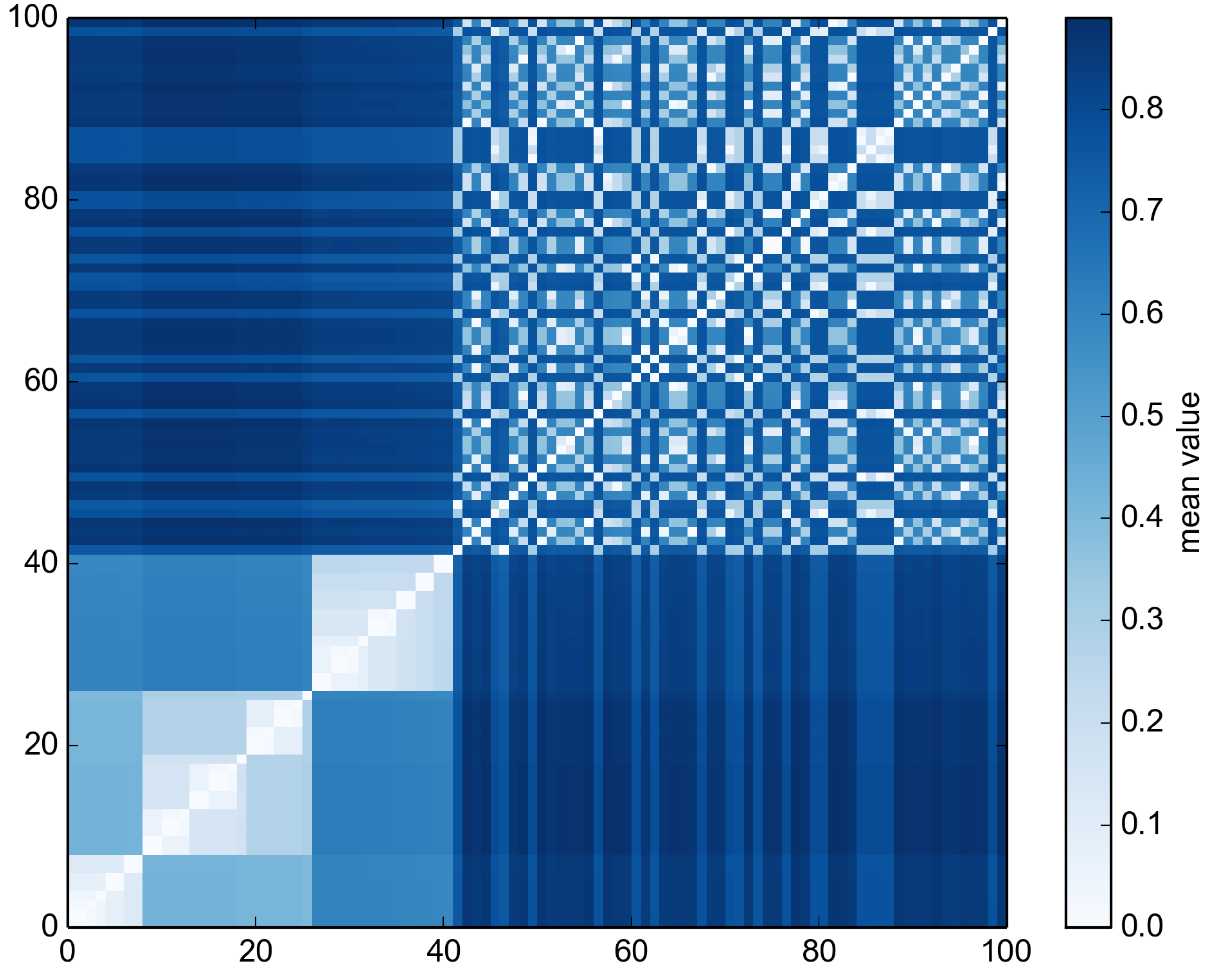

Figure 30: Heatmap of the pairwise “tree distance” matrix based on simulated species tree No. 5. Species 1-41 are “archaea”, species 42-100 are “bacteria”.

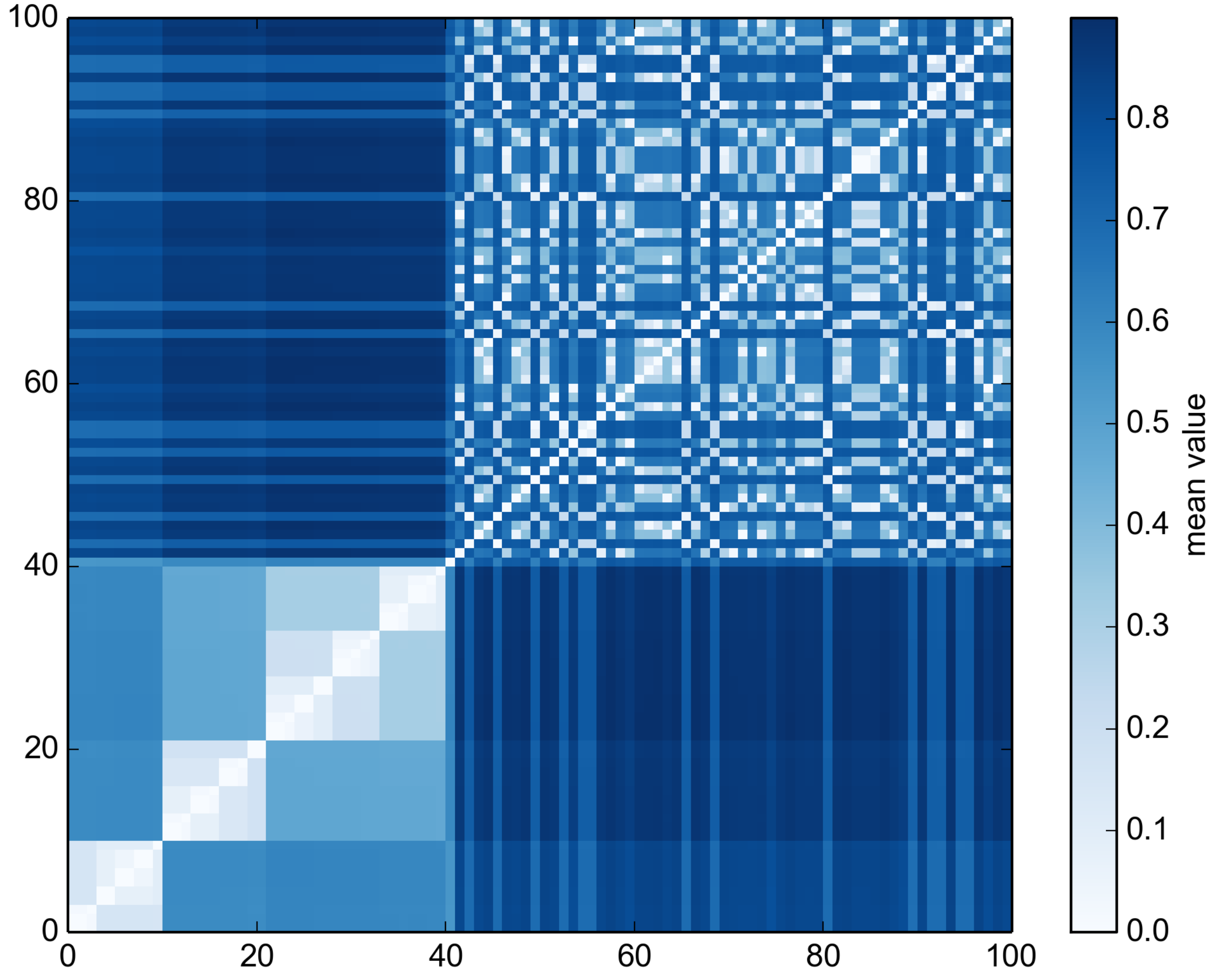

Figure 31: Heatmap of the pairwise "tree distance" matrix based on simulated species tree No. 6. Species 1-41 are "archaea", species 42-100 are "bacteria".

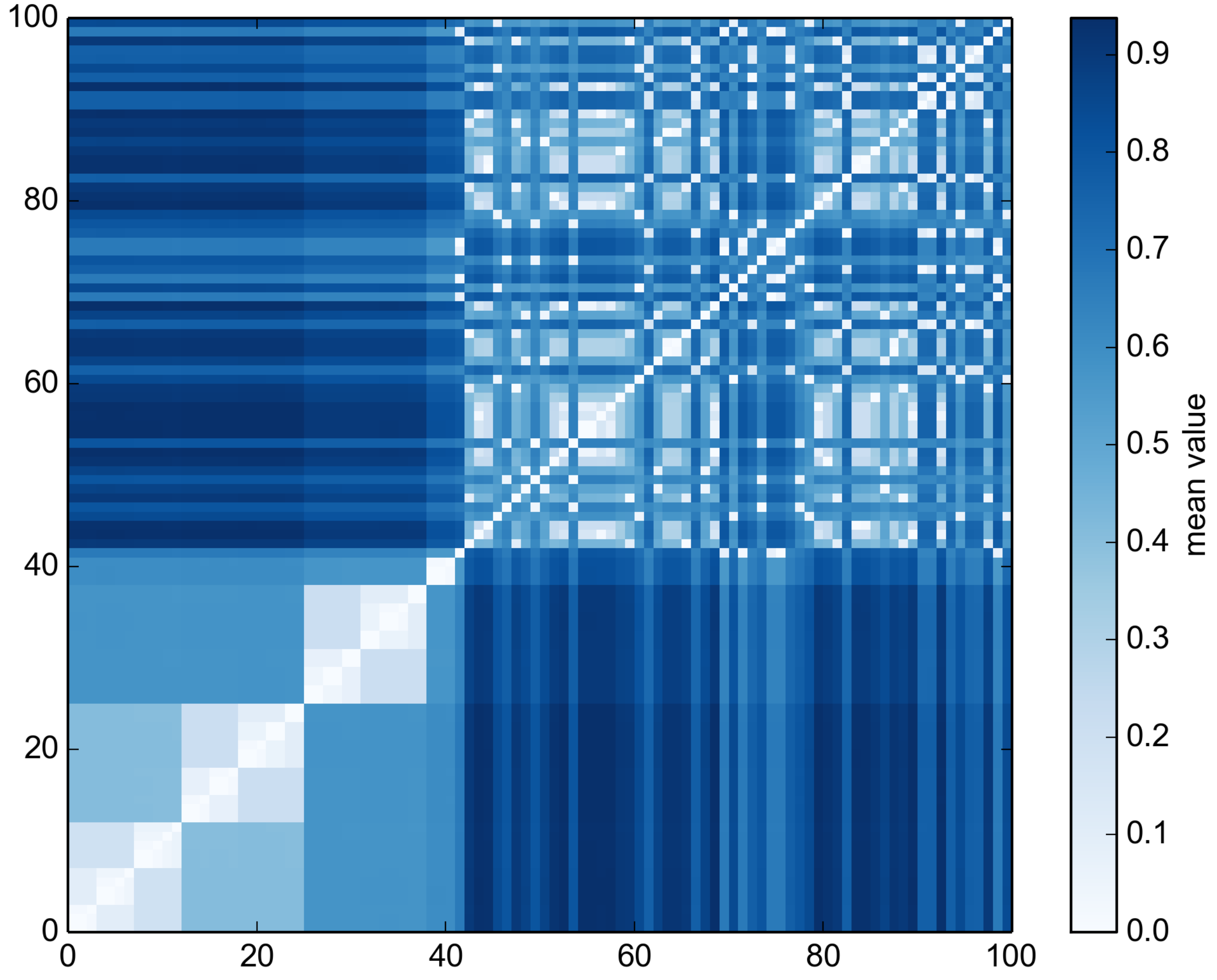

Figure 32: Heatmap of the pairwise "tree distance" matrix based on simulated species tree No. 7. Species 1-41 are "archaea", species 42-100 are "bacteria".

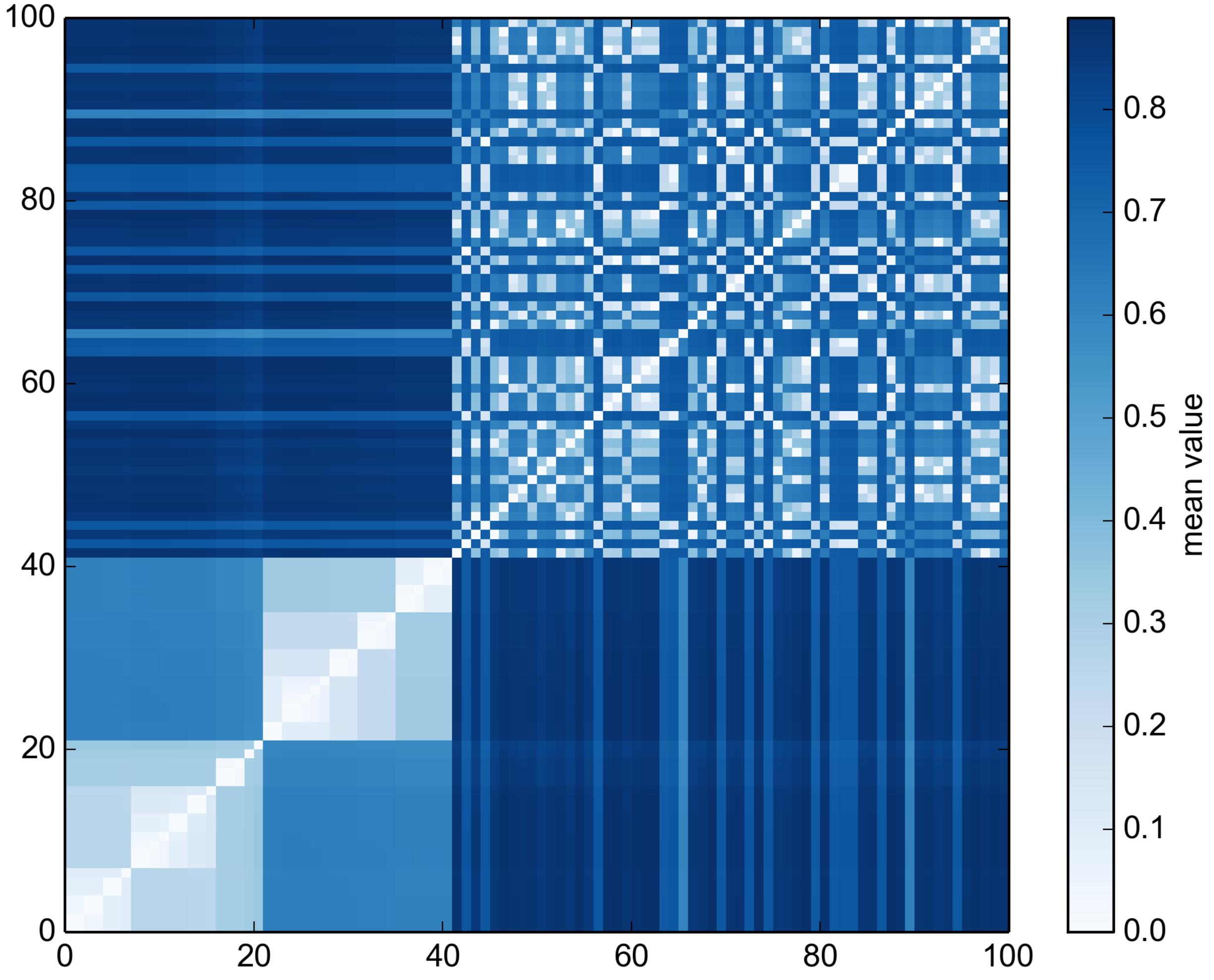

Figure 33: Heatmap of the pairwise “tree distance” matrix based on simulated species tree No. 8. Species 1-41 are “archaea”, species 42-100 are “bacteria”.

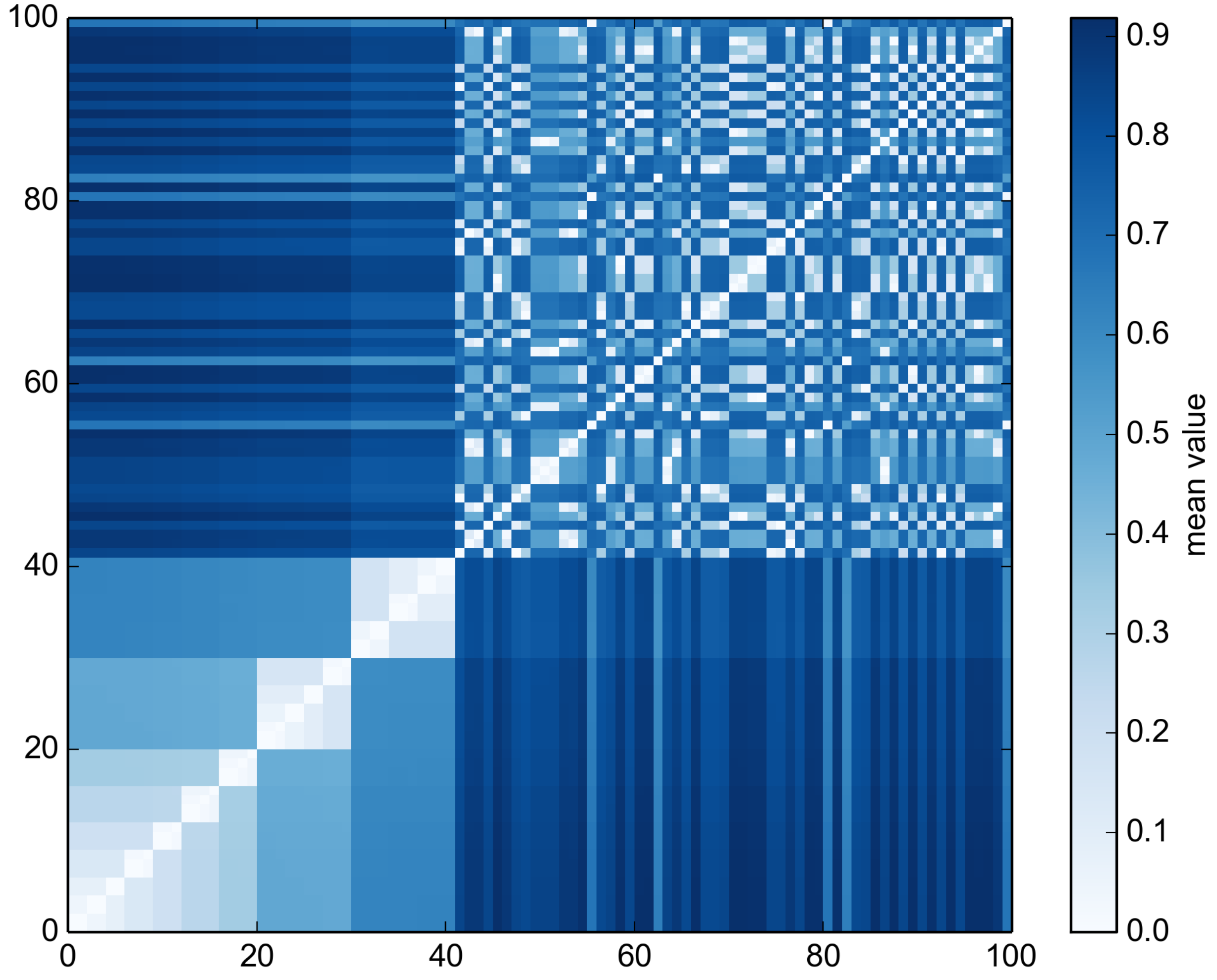

Figure 34: Heatmap of the pairwise "tree distance" matrix based on simulated species tree No. 9. Species 1-41 are "archaea", species 42-100 are "bacteria".

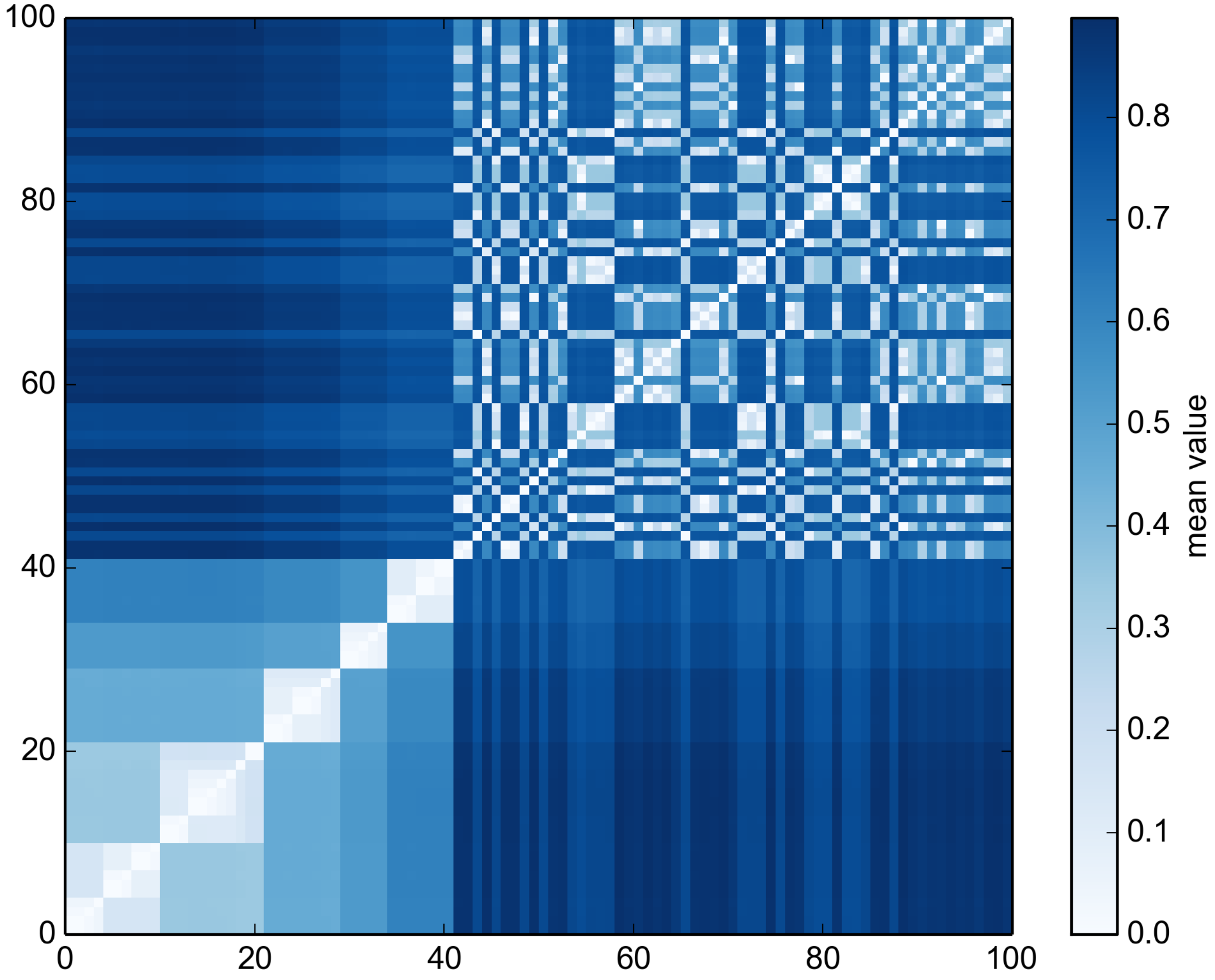

Figure 35: Heatmap of the pairwise "tree distance" matrix based on simulated species tree No. 10. Species 1-41 are "archaea", species 42-100 are "bacteria".

Appendix B: A figure of the QP2 tree

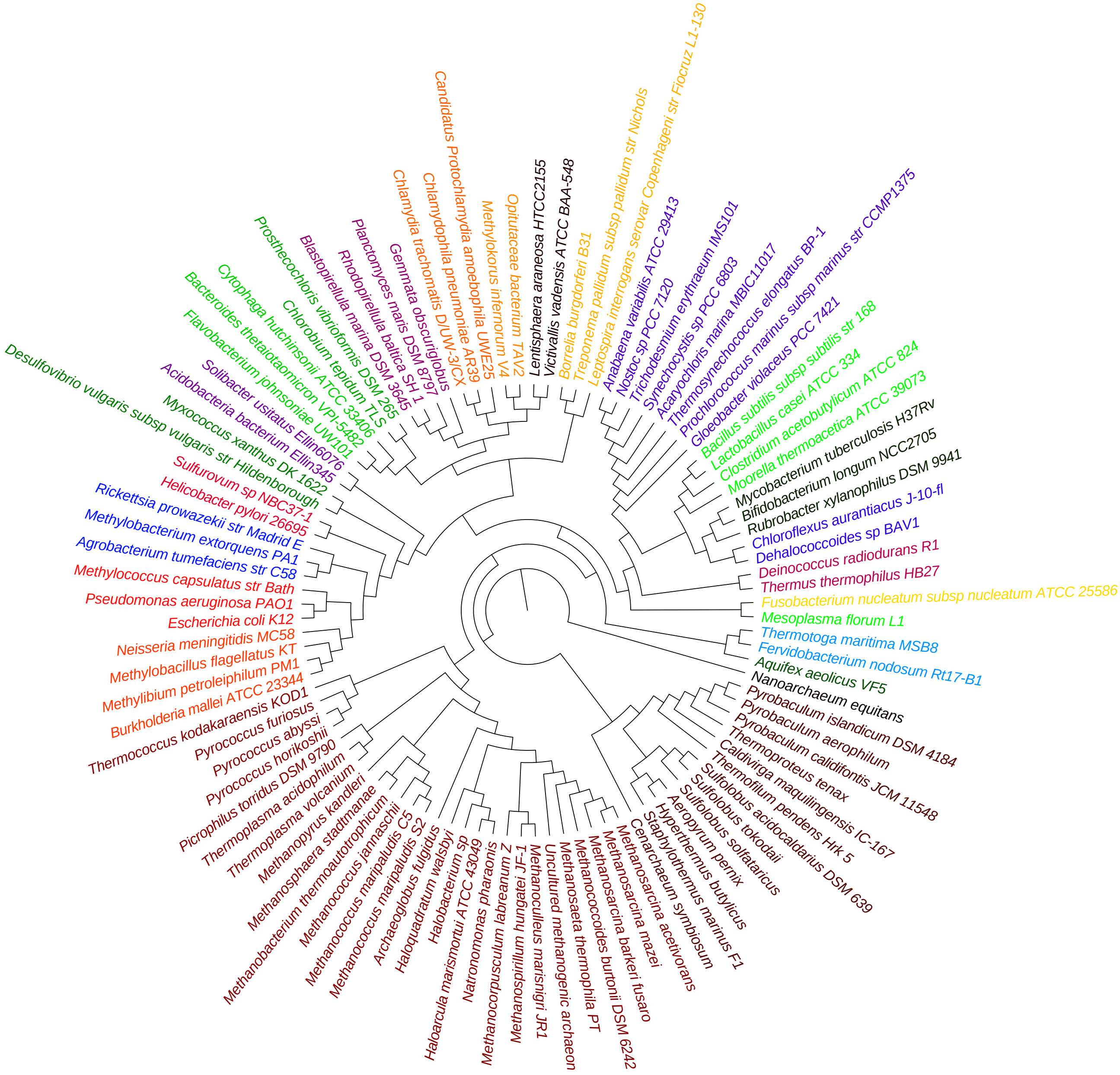

Appendix C: The QP1 tree

This is the QP1 tree in Newick format (Figure 9 in the main body of the text):

((((((((((('Methanosarcina acetivorans','Methanosarcina mazei'),'Methanosarcina barkeri fusaro'),'Methanococcoides burtonii DSM 6242'),'Methanosaeta thermophila PT'),'Uncultured methanogenic archaeon'),(('Methanoculleus marisnigri JR1','Methanospirillum hungatei JF-1'),'Methanocorpusculum labreanum Z')),((('Natronomonas pharaonis','Haloarcula marismortui ATCC 43049'),'Halobacterium sp'),'Haloquadratum walsbyi')),'Archaeoglobus fulgidus'),(((('Methanococcus maripaludis S2','Methanococcus maripaludis C5'),'Methanococcus jannaschii'),('Methanobacterium thermoautotrophicum','Methanosphaera stadtmanae')),'Methanopyrus kandleri')),((((((((('Pyrobaculum islandicum DSM 4184','Pyrobaculum aerophilum'),'Pyrobaculum calidifontis JCM 11548'),'Thermoproteus tenax'),'Caldivirga maquilingensis IC-167'),'Thermofilum pendens Hrk 5'),((('Sulfolobus acidocaldarius DSM 639','Sulfolobus tokodaii'),'Sulfolobus solfataricus'),(('Aeropyrum pernix','Hyperthermus butylicus'),'Staphylothermus marinus F1'))),'Nanoarchaeum equitans'),'Cenarchaeum symbiosum'),((('Pyrococcus horikoshii','Pyrococcus abyssi'),'Pyrococcus furiosus'),'Thermococcus kodakaraensis KOD1')),(('Thermoplasma volcanium','Thermoplasma acidophilum'),'Picrophilus torridus DSM 9790'))),((((((((((((('Burkholderia mallei ATCC 23344','Methylibium petroleiphilum PM1'),'Methylobacillus flagellatus KT'),'Neisseria meningitidis MC58'),(('Escherichia coli K12','Pseudomonas aeruginosa PAO1'),'Methylococcus capsulatus str Bath')),(('Agrobacterium tumefaciens str C58','Methylobacterium extorquens PA1'),'Rickettsia prowazekii str Madrid E')),'Desulfovibrio vulgaris subsp vulgaris str Hildenborough'),('Helicobacter pylori 26695','Sulfurovum sp NBC37-1')),(('Acidobacteria bacterium Ellin345','Solibacter usitatus Ellin6076'),'Myxococcus xanthus DK 1622')),((((('Blastopirellula marina DSM 3645','Rhodopirellula baltica SH 1'),'Planctomyces maris DSM 8797'),'Gemmata obscuriglobus'),((('Chlamydia trachomatis D/UW-3/CX','Chlamydophila pneumoniae AR39'),'Candidatus Protochlamydia amoebophila UWE25'),(('Lentisphaera araneosa HTCC2155','Victivallis vadensis ATCC BAA-

548'),('Methylokorus infernorum V4','Opitutaceae bacterium TAV2')))),(((('Cytophaga hutchinsonii ATCC 33406','Flavobacterium johnsoniae UW101'),'Bacteroides thetaiotaomicron VPI-5482'),('Chlorobium tepidum TLS','Prosthecochloris vibrioformis DSM 265')),'Leptospira interrogans serovar Copenhageni str Fiocruz L1-130'))),('Borrelia burgdorferi B31','Treponema pallidum subsp pallidum str Nichols')),(((((((((('Anabaena variabilis ATCC 29413','Nostoc sp PCC 7120'),'Trichodesmium erythraeum IMS101'),'Acaryochloris marina MBIC11017'),'Synechocystis sp PCC 6803'),'Thermosynechococcus elongatus BP-1'),'Prochlorococcus marinus subsp marinus str CCMP1375'),'Gloeobacter violaceus PCC 7421'),'Chloroflexus aurantiacus J-10-fl'),((('Mycobacterium tuberculosis H37Rv','Bifidobacterium longum NCC2705'),'Rubrobacter xylanophilus DSM 9941'),('Deinococcus radiodurans R1','Thermus thermophilus HB27'))),(((('Bacillus subtilis subsp subtilis str 168','Lactobacillus casei ATCC 334'),'Clostridium acetobutylicum ATCC 824'),'Moorella thermoacetica ATCC 39073'),'Dehalococcoides sp BAV1'))),'Mesoplasma florum L1'),'Fusobacterium nucleatum subsp nucleatum ATCC 25586'),(('Thermotoga maritima MSB8','Fervidobacterium nodosum Rt17-B1'),'Aquifex aeolicus VF5')));

Appendix D: The QP2 tree

This is the QP2 tree in Newick format. See also Appendix B.

((((((((((((('Methanosarcina acetivorans','Methanosarcina mazei'),'Methanosarcina barkeri fusaro'),'Methanococcoides burtonii DSM 6242'),'Methanosaeta thermophila PT'),'Uncultured methanogenic archaeon'),(('Methanoculleus marisnigri JR1','Methanospirillum hungatei JF-1'),'Methanocorpusculum labreanum Z')),((('Natronomonas pharaonis','Haloarcula marismortui ATCC 43049'),'Halobacterium sp'),'Haloquadratum walsbyi')),'Archaeoglobus fulgidus'),(((('Methanococcus maripaludis S2','Methanococcus maripaludis C5'),'Methanococcus jannaschii'),('Methanobacterium thermoautotrophicum','Methanosphaera stadtmanae')),'Methanopyrus kandleri')),(('Thermoplasma volcanium','Thermoplasma acidophilum'),'Picrophilus torridus DSM 9790')),((('Pyrococcus horikoshii','Pyrococcus abyssi'),'Pyrococcus furiosus'),'Thermococcus kodakaraensis KOD1')),(((((((('Pyrobaculum islandicum DSM 4184','Pyrobaculum aerophilum'),'Pyrobaculum calidifontis JCM 11548'),'Thermoproteus tenax'),'Caldivirga maquilingensis IC-167'),'Thermofilum pendens Hrk 5'),((('Sulfolobus acidocaldarius DSM 639','Sulfolobus tokodaii'),'Sulfolobus solfataricus'),(('Aeropyrum pernix','Hyperthermus butylicus'),'Staphylothermus marinus F1'))),'Cenarchaeum symbiosum'),'Nanoarchaeum equitans')),((((((((((('Burkholderia mallei ATCC 23344','Methylibium petroleiphilum PM1'),'Methylobacillus flagellatus KT'),'Neisseria meningitidis MC58'),(('Escherichia coli K12','Pseudomonas aeruginosa PAO1'),'Methylococcus capsulatus str Bath')),(('Agrobacterium tumefaciens str C58','Methylobacterium extorquens PA1'),'Rickettsia prowazekii str Madrid E')),('Helicobacter pylori 26695','Sulfurovum sp NBC37-1')),('Desulfovibrio vulgaris subsp vulgaris str Hildenborough','Myxococcus xanthus DK 1622')),('Acidobacteria bacterium Ellin345','Solibacter usitatus Ellin6076')),((((('Flavobacterium johnsoniae UW101','Bacteroides thetaiotaomicron VPI-5482'),'Cytophaga hutchinsonii ATCC 33406'),('Chlorobium tepidum TLS','Prosthecochloris vibrioformis DSM 265')),(((('Blastopirellula marina DSM 3645','Rhodopirellula baltica SH 1'),'Planctomyces maris DSM 8797'),'Gemmata obscuriglobus'),((('Chlamydia tra-

chomatis D/UW-3/CX','Chlamydophila pneumoniae AR39'),'Candidatus Protochlamydia amoebophila UWE25'),(('Methylokorus infernorum V4','Opitutaceae bacterium TAV2'),('Lentisphaera araneosa HTCC2155','Victivallis vadensis ATCC BAA-548')))))),(('Borrelia burgdorferi B31','Treponema pallidum subsp pallidum str Nichols'),'Leptospira interrogans serovar Copenhageni str Fiocruz L1-130'))),((((((((('Anabaena variabilis ATCC 29413','Nostoc sp PCC 7120'),'Trichodesmium erythraeum IMS101'),'Synechocystis sp PCC 6803'),'Acaryochloris marina MBIC11017'),'Thermosynechococcus elongatus BP-1'),'Prochlorococcus marinus subsp marinus str CCMP1375'),'Gloeobacter violaceus PCC 7421'),(((('Bacillus subtilis subsp subtilis str 168','Lactobacillus casei ATCC 334'),'Clostridium acetobutylicum ATCC 824'),'Moorella thermoacetica ATCC 39073'),((('Mycobacterium tuberculosis H37Rv','Bifidobacterium longum NCC2705'),'Rubrobacter xylanophilus DSM 9941'),('Chloroflexus aurantiacus J-10-fl','Dehalococcoides sp BAV1')))),('Deinococcus radiodurans R1','Thermus thermophilus HB27')),('Fusobacterium nucleatum subsp nucleatum ATCC 25586','Mesoplasma florum L1'))),(('Thermotoga maritima MSB8','Fervidobacterium nodosum Rt17-B1'),'Aquifex aeolicus VF5')));
